# CircRNA-14052 promotes breast cancer progression via miR-214-3p/IKBKB pathway

**DOI:** 10.1186/s41065-025-00566-6

**Published:** 2025-10-03

**Authors:** Donghai Li, Zhiying Zhang, Yan Cui, Jiaxin Sun, Wenyuan Wei, Shaofeng Yang, Rui Zhang

**Affiliations:** 1 Department of Thyroid Breast Surgery, Affiliated Hospital of Inner Mongolia Medical University, Hohhot North Street, Hohhot, 010050 Inner Mongolia China; 2 School of Humanities Education, Inner Mongolia Medical University, Huhhot, 010000 Inner Mongolia China; 3 Clinical Medical Research Center, Affiliated Hospital of Inner Mongolia Medical University, Hohhot North Street, Hohhot, 010050 Inner Mongolia China

**Keywords:** Breast cancer, CircRNA, circRNA-14052, miR-214-3p, IKBKB, JAK2-STAT3

## Abstract

**Objectives:**

Circular RNAs play crucial regulatory roles in the progression of human diseases. This study aimed to investigate the functional mechanism of circRNA-14,052 in breast cancer progression.

**Methods:**

The biological functions of circRNA-14,052 were assessed using CCK-8, wound healing, flow cytometry assays. The ceRNA regulatory network of circRNA-14,052-miR-214-3p- IKBKB was validated by luciferase reporter assay.

**Results:**

The levels of circRNA-14,052 were notably elevated, but the levels of miR-214-3p were markedly reduced in breast cancer tissues compared to adjacent non-cancerous tissues. Downregulation of circRNA-14,052 or overexpression of miR-214-3p reduced MCF-7 cell proliferation and triggered cell apoptosis. Mechanically, circRNA-14,052 could elevate IKBKB levels via competitively sponging miR-214-3p. Notably, miR-214-3p inhibition reversed the growth-suppressive effects of circRNA-14,052 silencing. Additionally, circRNA-14,052 knockdown notably reduced IKBKB, IL-6, JAK2 and STAT3 levels in MCF-7 cells; whereas these changes were reversed by miR-214-3p deficiency. Furthermore, deficiency of circRNA-14,052 reduced xenograft tumor growth in vivo through targeting miR-214-3p/IKBKB/IL-6/JAK2/STAT3 axis.

**Conclusion:**

Collectively, our results showed that circRNA-14,052 promotes breast cancer progression via the miR-214-3p/IKBKB axis. Targeting this molecular axis may represent a promising therapeutic strategy for breast cancer treatment.

**Supplementary Information:**

The online version contains supplementary material available at 10.1186/s41065-025-00566-6.

## Introduction

Breast cancer has become one of the most frequent malignant diseases among global women [1]. It can be classified into four major molecular subtypes: Luminal A, Luminal B, HER2-enriched breast cancer, and triple-negative breast cancer (TNBC) [2]. Despite significant progress in standardized treatment, 30%-40% of early breast cancer patients eventually develop metastasis [1]. Given its substantial impact on public health, understanding the molecular mechanisms underlying breast cancer pathogenesis remains crucial. Thus, we tried to explore the molecular mechanism and found new molecular targets for the treatment and prognosis of breast cancer.

Circular RNAs (circRNAs) widely exist in eukaryotic cells with highly conserved gene sequences and stable molecular structure [3]. As nonlinear ribonucleic acid, circRNA forms a continuous loop through back-splicing. Early research suggested that circRNAs have no biological function but are a byproduct of mis-splicing during messenger RNA processing [4]. Currently, circRNAs are attached more and more importance as a novel class of non-coding RNAs. Many studies have shown that circRNAs interact with micro RNAs (miRNAs) to regulate related genes’ levels in the signaling pathway, thereby affecting tumor growth, and drug resistance [5–7]. The circRNA-miRNA axis is believed to be a critical target for tumor treatment and has great potential in the diagnosis of cancers.

Our results indicate that circRNA-14,052 levels are notably increased in breast cancer tissues compared to adjacent non-cancerous tissues. Given its marked dysregulation and unknown biological functions, we then aimed to explore the functional role and molecular mechanisms of circRNA-14,052 in breast cancer. We identified miR-214-3p as the binding molecule of circRNA-14,052, noting that their interaction may influence tumor cell growth. MiR-214-3p has emerged as a significant tumor suppressor in various cancers, including breast cancer [8, 9]. However, the specific mechanism by which circRNA-14,052 mediates its effects in breast cancer remains unclear. In this study, we found that circRNA-14,052 may promote breast cancer progression through the sponging of miR-214-3p, leading to subsequent dysregulation of the IKBKB pathway. Altogether, our findings reveal circRNA-14,052 as a tumor promoter, suggesting that it may serve as a potential therapeutic target for breast cancer.

## Materials and methods

### Clinical samples

Nine pairs of tumor tissues and adjacent normal tissues were collected from three breast cancer patients who had surgeries at Affiliated Hospital of Inner Mongolia Medical University. The criteria for inclusion included: (a) all subjects satisfied the diagnostic requirements for breast cancer; (b) complete clinical and imaging records were available; (c) comprehensive clinicopathological data for each patient was provided; (d) patients with a history of other malignancies were excluded. The ethical committee of Inner Mongolia Medical University (grant number YJS2024055) approved this study, and all participants gave their informed consent, adhering to the Declaration of Helsinki. Supplementary Table S1 summarize patient clinical information.

### Realtime quantitative PCR

Total RNA was extracted with the use of Redzol Reagent (FTR-50, Sbsgenetech, Beijing, China) and examined by Nanodrop (Biosystems, Germany). The extracted RNA underwent reverse transcription to form cDNAs utilizing SureScript™ First-Strand cDNA Synthesis Kit (QP056, GeneCopeia, US). RT-qPCR was conducted using the 2×SYBR Green qPCR Master Mix (MPC2203026, Servicebio, Wuhan, China). For mRNA, GAPDH served as the internal reference, whereas U6 gene was used for miRNA. Each sample had three replicates. The expression levels were calculated by the 2^–△△CT^ method. All primer sequences were shown in Table 1.

**Table 1:** Primer sequences for RT-qPCR

Genes	Forward Primer (5’-3’)	Reverse Primer (5’-3’)
GAPDH	CGAGATCCCTCCAAAATCAA	TTCACACCCATGACGAACAT
IKBKB	TGAGAAGACTGTTGTCCGGC	GCAGGGTGCAGAGGTTATGT
miR-214-3p	AGCAGGCACAGACAGGCA	GCAGGGTCCGAGGTATTC
circRNA-14,052	TTGCATGCTCTAACCCTGGG	TACCCCTTATCTGCCAGCCT
U6	CTCGCTTCGGCAGCACA	AACGCTTCACGAATTTGCGT
IL-6	TGCCTTCTTGGGACTGAT	CTGGCTTTGTCTTTCTTGTT
JAK2	AATGAGTGAAACCGAAAG	CAGTACCAATGAGGGAAG
STAT3	CACCCAACAGCCGCCGTAGT	TGCCGCCTCTTCCAGTCA

### Cell culture and transfection

Human breast cancer cell lines MCF-7 (BNCC100137, BeNa Culture Collection), MDA-MB-231 (BNCC337894, BeNa Culture Collection) and HEK293T cells (CL-0005, Pricella) were utilized at low passage numbers (< 10) [10]. MCF-7 cells were cultured in DMEM supplemented with 1% penicillin/streptomycin, 0.01 mg/ml insulin and 10% fetal bovine serum (FBS) at 37˚C with 5% CO_2_. MDA-MB-231 and HEK293T cells were cultured in DMEM supplemented with 1% penicillin/streptomycin and 10% FBS at 37˚C with 5% CO_2_.

For transient transfection, MCF-7 and MDA-MB-231 cells were transfected with miR-214-3p mimic or inhibitor using Lipofectamine 2000 transfection reagent.

### Construction of stable cell lines

The sequence of circRNA14052 siRNA (sense: 5’-UUUAUACCACACUCAAACCCU-3’; anti-sense: 5’-GGUUUGAGUGUGGUAUAAAUU-3’) was cloned into the PGMLV-6751 lentiviral vector. CircRNA14052 overexpression (circRNA-14052-OE) plasmids were obtained from Hanbio. The empty vector was used as the negative control. These vectors were then transfected into HEK293T cells using the Lipofectamine 2000. After 72 h, the virus were collected and transduced into breast cancer cells. After 72 h of cultivation, cells were selected using a culture medium containing puromycin. Two weeks later, a stable circRNA-14,052 knockdown cell line (LV-circRNA-14052 siRNA) and a stable circRNA-14,052 overexpression cell line (LV-circRNA-14052 OE) were established.

### Cell counting kit-8 (CCK-8) assay

Cell viability was evaluated using CCK-8 (C0009S, Beyotime) reagent. Cells were seeded in a 96-well plate and cultured in a 37℃ incubator. At the time points of 24, 48, and 72 h, all cells were washed by HBSS and added fresh medium with 10 µL of CCK-8 solution for 4 h at 37 ℃. The optical density (OD) of each well was measured at 450 nm using a microplate reader.

### Colony-formation assay

Cells were seeded on a 12-well plate, with 1000 cells placed in each well and 3 replicates set for each group. The cells were then incubated for a week until colony formation was visible. The culture medium was discarded and replaced with cell fixative (BN20094, Bioregenmed) to fix for 30 min. After removing the cell fixative, the samples were stained with 0.1% crystal violet staining solution for 3 min. The colonies were observed using a microscope and subsequently counted.

### Flow cytometry

Flow cytometry was performed according to the instructions of Annexin V-FITC/PI apoptosis kit (AP101, Liankebio). The cells were suspended and mixed with Annexin V-FITC (5 µl). Then, propidium iodide (PI, 5 µl) staining solution was added and incubated in the dark for 15 min, followed by an ice bath. Experiments were performed on a flow cytometer (BD Biosciences) with three replicates set for each group.

### Dual-luciferase reporter assay

The dual-luciferase reporter was modified by cloning either the wild-type or mutant 3’-UTR of IKBKB or the wild-type or mutant form of circRNA14052, which contains the binding site for miR-214-3p. Next, 293T cells underwent co-transfection with either miR-214-3p mimics or inhibitor, along with the aforementioned dual-luciferase reporter plasmids, using Lipofectamine 2000 (Invitrogen) for 48 h. The Dual-luciferase Gene Detection Kit (RG006, Beyotime) was performed to assess the luciferase activity.

### Transwell invasion assay

Matrigel (Corning, 356234) was diluted with DMEM to a concentration of 300 µg/mL on ice and then 100 µL was applied to coat the surface of the membrane in the chamber. The chamber was subsequently placed in a 24-well plate and incubated for three hours at 37 ℃. In the lower chamber, 600 µL of medium supplemented with 10% FBS was added. Each well in the upper chamber received 100 µL of a cell suspension containing 1*10^4^ cells, which were allowed to incubate for 24 h. After that, the cells were stained with 0.1% crystal violet for one hour. A microscope was utilizing for capturing images of the invasive cells.

### Wound-healing assay

5 * 10^5^ cells were placed on a 6-well plate and cultured in a 5% CO_2_ incubator at 37 ℃. Once the cell density reached approximately 90% or more, each well was scratched with the tip of a sterile pipette. Photographs were taken at 0 h, 24 h, and 48 h post-scratch. The wound-healing results were analyzed using Image J software.

### Western blot

Western blot assay was employed to determine the protein levels of STAT3, JAK2, IL-6 and IKK beta (IKBKB) in MCF-7 cells or tumor tissues. This method consists of four main steps: (Ⅰ) protein extraction and quantification; (Ⅱ) electrophoresis and transfer; (Ⅲ) immunoblotting and (Ⅳ) detection and analysis. First, proteins were extracted from cells using RIPA lysis buffer (BN25001-A, Bioregenmed, China) and quantified by a BCA test kit (Utibody, SL201). Second, the protein samples were loaded onto 5% SDS-PAGE gel and subsequently transferred onto a PVDF membrane (0000175594, Millipore). Third, the PVDF membrane was blocked in 5% skim milk for 2 h at room temperature, and then incubated with primary antibodies (anti-STAT3 antibody, ab68153, 1:1000; anti-JAK2 antibody, ab108596, 1:2500; anti-IL-6 antibody, ab233551, 1:1000; anti-IKK beta (IKBKB) antibody, ab32135, 1:1000) overnight at 4 ℃ and with a secondary antibody (goat anti-rabbit IgG H&L (HRP), ab205718, 1:8000, Abcam) for 1.5 h. Finally, images were captured using a chemiluminescence imaging system (ChemiScope6100) and analyzed for grayscale using Image J software.

### Immunofluorescence staining assay

The cells were fixed with 4% paraformaldehyde fixative (P0099, Beyotime) for 40 min and washed with PBS three times. The samples were blocked by BCA for 90 min and washed twice with PBS. Samples were subjected to probing with primary antibodies (anti-IL-6 antibody, ab233551; anti-JAK2 antibody, ab108596; anti-STAT3 antibody, ab68153; Abcam), and then a secondary antibody was applied for 20 min. Images were photographed under a fluorescence microscope (BH2-RFCA, OLYMPUS). The nucleus were stained with DAPI (C0065, Solarbio, Beijing, China).

### Animal experiments

#### Ethical approval and animal care

Experiments involving animals were performed in compliance with guidelines by the National Institutes of Health Guidelines for Care and Use of Laboratory Animals and in alignment with ARRIVE guidelines. Animal experiments were approved by the ethics committee of Inner Mongolia Medical University (YJS2024055). A total of twenty BALB/c nude mice (SPF-level, purchased from Spfbiotech, Beijing, China) were fed in a place with a standard light-dark cycle at room temperature (21–26℃) and humidity of 50–60%, with food and water ad libitum.

#### Experimental groups and tumor implantation

After one-week acclimatization, all mice were randomly divided into four groups (*n* = 5/group) [11]: Control, LV-NC, LV-circRNA-14,052-OE, and LV-circRNA-14,052-siRNA. 1*10^6^ MCF-7 cells or lentivirus-transfected cells were subcutaneously injected into the right axilla of mice.

#### Tumor growth monitoring

The width and weight of the tumors were recorded once a week for five weeks in a blinded manner. The tumor volume was calculated using the formula: (length × width²)/2. After 35 days, all mice were sacrificed by cervical dislocation under anesthesia (1% isoflurane inhalation), followed by isolation of the tumors. Mice were humanely euthanized if they reached humane endpoints, such as tumors exceeding 2 cm (no animals met these criteria) [12, 13].

### Histopathology study

After dewax and rehydrate, the tumor sections were stained by Harris’ hematoxylin (ZLI-9610, Zsbio, Beijing, China) for 5–10 min, followed by staining with eosin (ZLI-9613, Zsbio, Beijing, China) for 1–3 min. The sections were dehydrated by alcohol until transparent, sealed with neutral gum, and then observed under a microscope (CKX43-LP, OLYMPUS, Japan).

### Terminal Deoxynucleotidyl transferase (TdT) dUTP Nick-End labeling (TUNEL) staining assay

Cell apoptosis was assessed using the TUNEL kit (G1504, Servicebio). Briefly, sections were incubated with CF488 dUTP Labeling Mix and Recombinant TDT enzyme at 37 °C for one hour. Next, sections were stained with DAPI for 5 min. A fluorescence microscope was used for observing cell apoptosis in tumor tissues.

### Statistical analysis

All data were presented as the mean ± standard deviation (SD). A t-test was employed to compare the differences in circRNA14052 and miR-214-3p levels between tumor and matching normal tissues. Two-way analysis of variance (ANOVA) was utilized to analyze the differences in tumor growth curves and body weight data. The dual-luciferase reporter gene assay and CCK-8 assay were also analyzed using two-way ANOVA. For other experiments, one-way ANOVA was applied to determine differences among multiple groups. All analyses were conducted using GraphPad Prism 9.0. At least three independent repeats were carried out for each experiment [14, 15]. A *p*-value < 0.05 was considered statistically significant.

## Results

### Overexpression of circRNA-14,052 promotes the proliferation of breast cancer cells

**Fig. 1:**
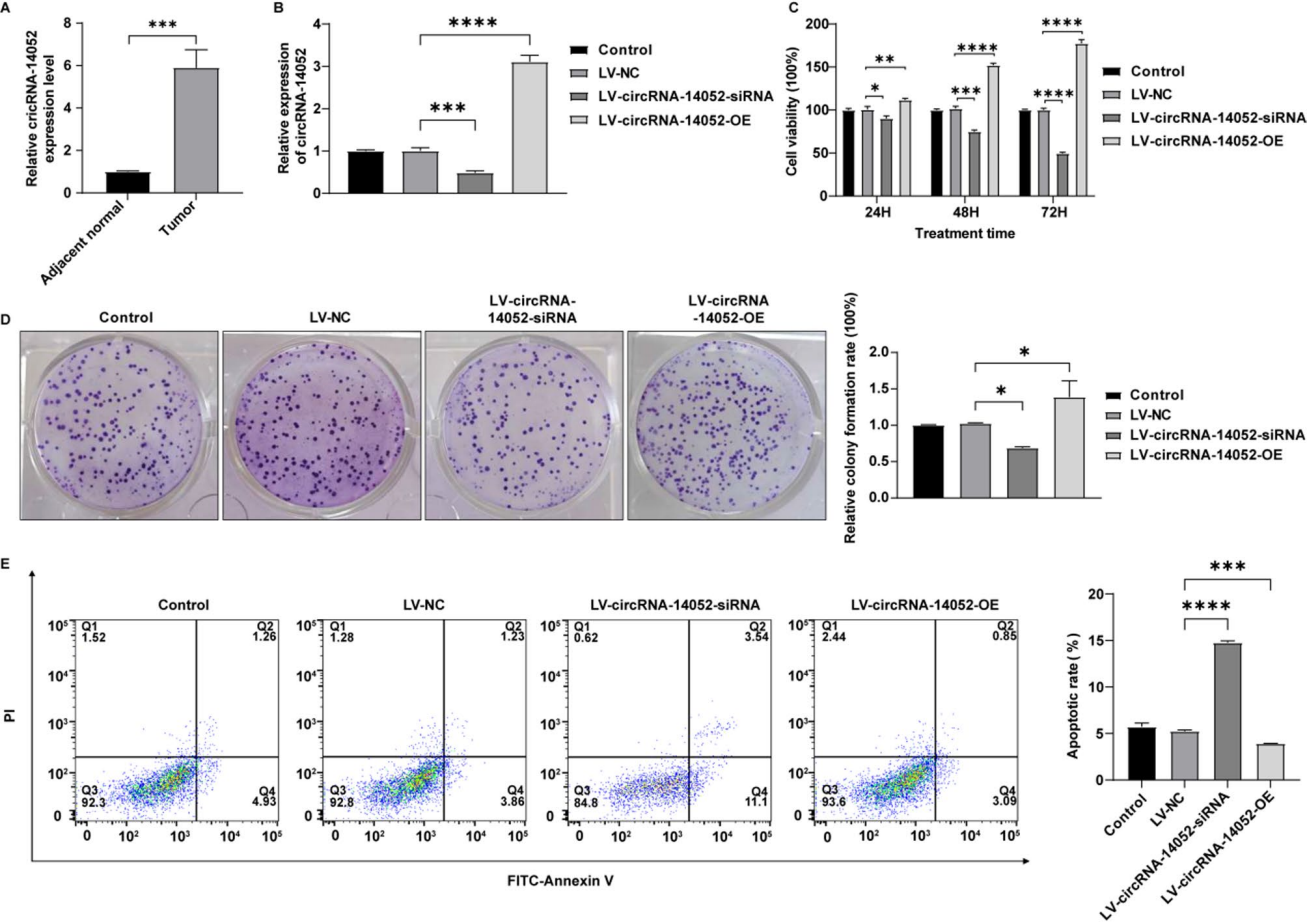
The role of circRNA-14,052 in breast cancer cells. (**A**) The levels of circRNA-14,052 were evaluated in adjacent non-cancerous tissues and breast cancer tissues by RT-qPCR (*n* = 9; t-test; **P < 0.01). (**B**) MCF-7 cells were transfected with LV-NC, LV-circRNA-14,052-siRNA and LV-circRNA-14,052-OE. The expression levels of circRNA-14,052 in MCF-7 cells were detected by RT-qPCR (*n* = 3; one-way ANOVA; ***P < 0.001, ****P < 0.0001). (**C**) The MCF-7 cell viability rate was detected by CCK-8 assay (*n* = 3; two-way ANOVA; *P<0.05, **P < 0.01, ***P < 0.001, ****P < 0.0001). (**D**) Cell proliferation was assessed by colony formation assay (*n* = 3; one-way ANOVA; *P < 0.05). (**E**) The apoptotic rate of MCF-7 cells was detected by flow cytometry (*n* = 3; one-way ANOVA; ***P < 0.001, ****P < 0.0001)

To determine the role of circRNA-14,052 in breast cancer, we first examined its expression in tumor samples collected from patients with breast cancer. Compared to adjacent non-cancerous tissues, circRNA-14,052 levels were notably elevated in breast cancer tissues (Fig. 1A). To characterize functionally the role of circRNA-14,052 in breast cancer, we overexpressed circRNA-14,052 using LV-circRNA-14,052-OE plasmid and knocked down circRNA-14,052 using LV-circRNA-14,052-siRNA plasmid in MCF-7 cells. CircRNA-14,052 levels were significantly increased in MCF-7 cells after transfection with LV-circRNA-14,052-OE plasmid, while the expression of circRNA-14,052 was significantly reduced in MCF-7 cells transfected with LV-circRNA-14,052-siRNA plasmids (Fig. 1B). Furthermore, knockdown of circRNA-14,052 remarkably reduced MCF-7 cell viability and proliferation, and increased cell apoptosis (Fig. 1C-E). Conversely, circRNA-14,052 overexpression exhibited the opposite effects (Fig. 1C-E). These results indicate that circRNA-14,052 exerts oncogenic functions in breast cancer.

### The negative regulatory effect of circRNA-14,052 on miR-214-3p

**Fig. 2:**
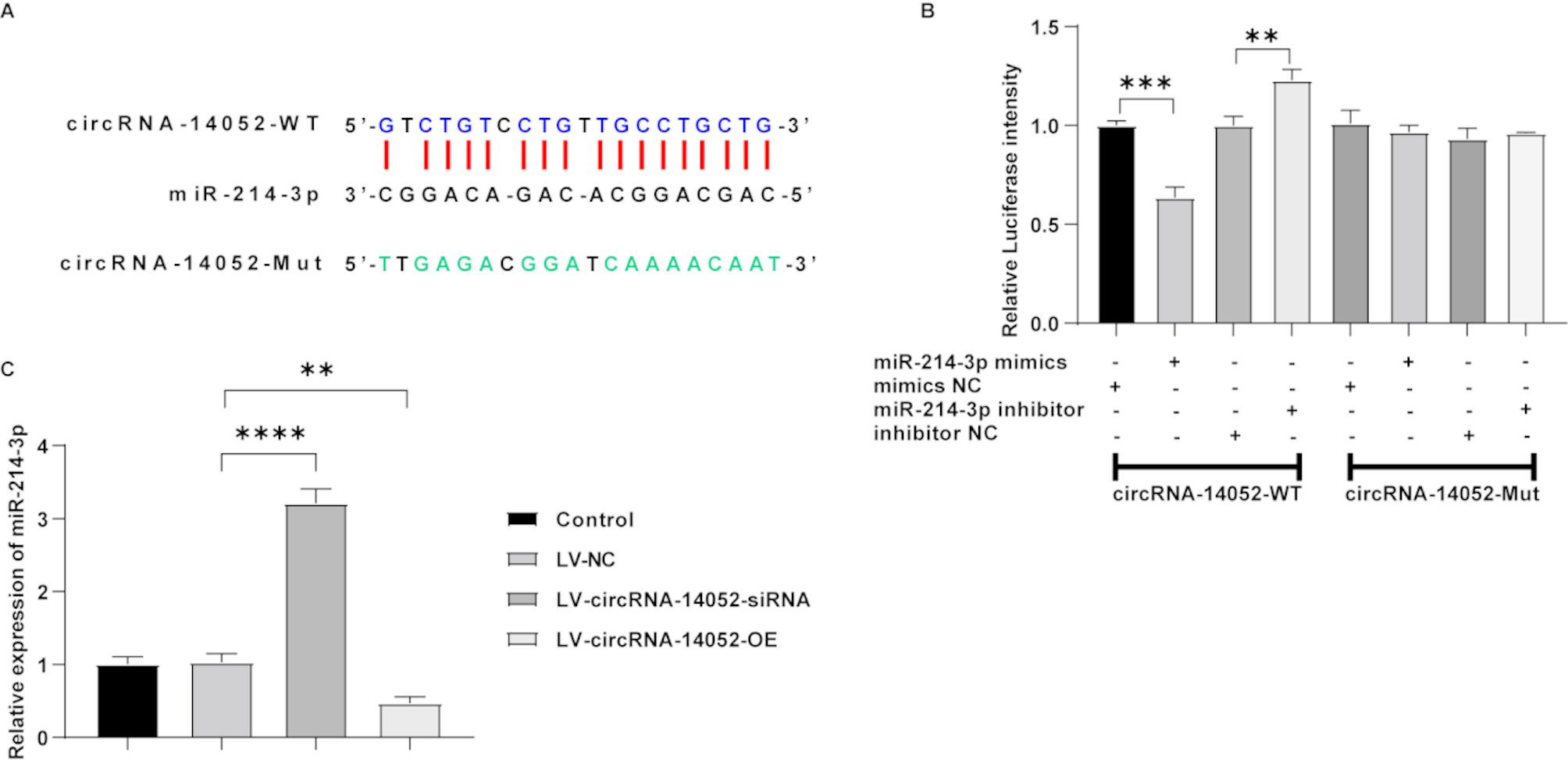
MiR-214-3p is a target of circRNA-14,052. (**A**) The predicted binding site between circRNA-14,052 and miR-214-3p. (**B**) The interaction between circRNA-14,052 and miR-214-3p was verified by dual-luciferase reporter assay (*n* = 3; two-way ANOVA; **P < 0.01, ***P < 0.001). (**C**) RT-qPCR analysis of miR-214-3p levels in MCF-7 cells transfected with LV-NC, LV-circRNA-14,052-siRNA or LV-circRNA-14,052-OE (*n* = 3; one-way ANOVA; **P < 0.01, ****P < 0.0001)

Based on the ceRNA hypothesis, circRNA may function as a competing endogenous RNA, influencing the expression of target genes of miRNA [16]. To explore the potential molecular mechanisms underlying the role of circRNA-14,052 in breast cancer progression, we identified a potential miRNA that could bind with circRNA-14,052. As shown in Fig. 2A, there was a binding site between miR-214-3p and circRNA-14,052. Moreover, the results of dual-luciferase reporter assay showed that miR-214-3p mimics significantly lowered the luciferase activity of the wild type of circRNA-14,052, while inhibition of its expression exhibited the opposite effects (Fig. 2B). These data suggested that circRNA-14,052 could directly bind with the miR-214-3p. Additionally, deficiency of circRNA-14,052 resulted in an obvious increase in miR-214-3p levels in MCF-7 cells, whereas overexpression of circRNA-14,052 led to an obvious decrease in miR-214-3p levels in MCF-7 cells (Fig. 2C). These suggest that circRNA-14,052 negatively regulates the expression of miR-214-3p.

### Overexpression of miR-214-3p suppresses the proliferation of breast cancer cells

**Fig. 3:**
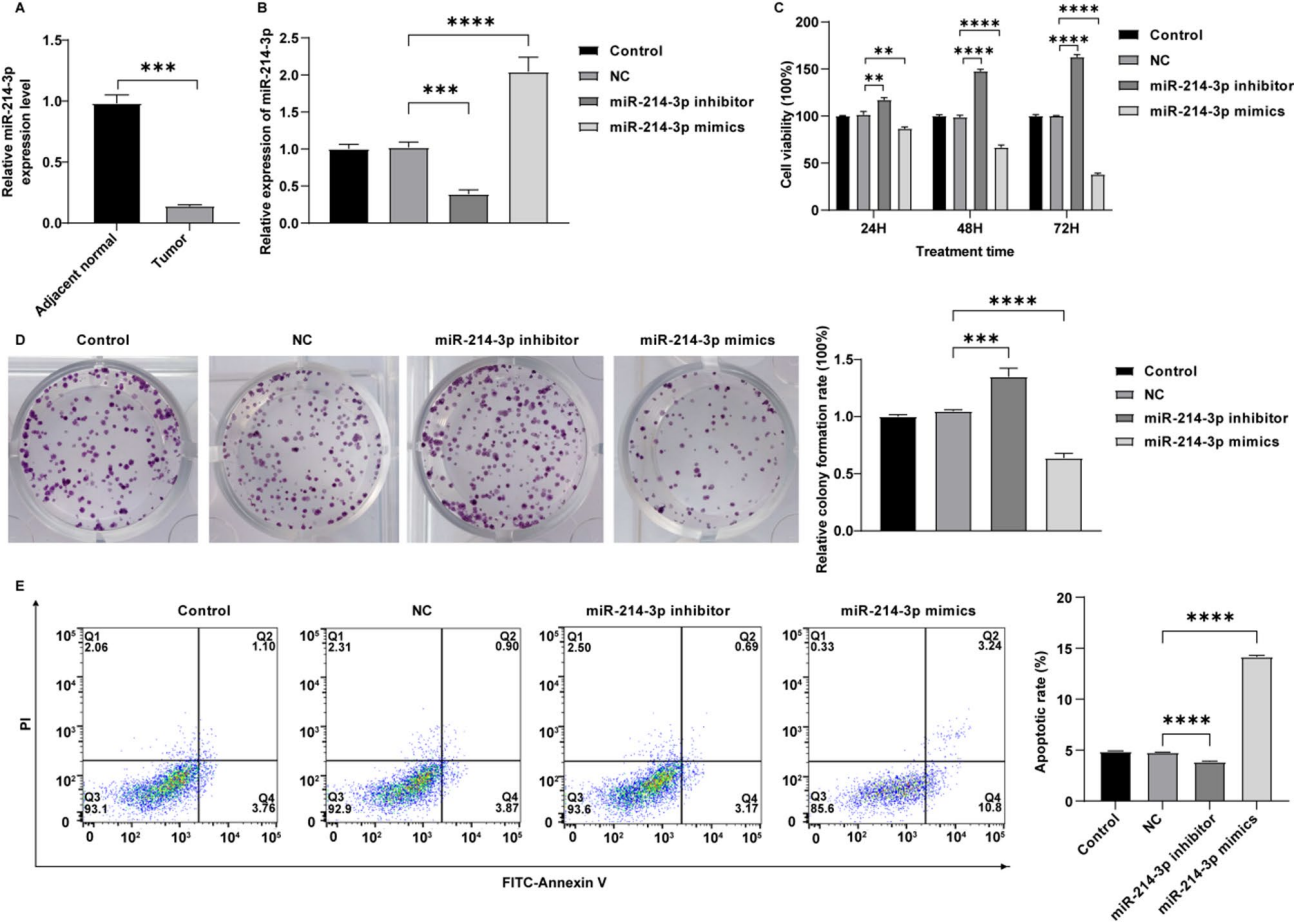
The role of miR-214-3p in breast cancer cells. (**A**) The levels of miR-214-3p were evaluated in adjacent non-cancerous tissues and breast cancer tissues by RT-qPCR (*n* = 9; t-test; ***P < 0.001). (**B**) MCF-7 cells were transfected with NC, miR-214-3p-mimics or miR-214-3p-inhibitor. The expression level of miR-214-3p in MCF-7 cells was detected by RT-qPCR (*n* = 3; one-way ANOVA; ***P < 0.001, ****P < 0.0001). (**C**) Cell viability was detected by CCK-8 assay (*n* = 3; two-way ANOVA; **P < 0.01, ****P < 0.0001). (**D**) Cell proliferation was detected by the colony formation assay (*n* = 3; one-way ANOVA; ***P < 0.001, ****P < 0.0001). (**E**) The apoptotic rate of the cancer cells was detected by flow cytometry (*n* = 3; one-way ANOVA; ****P < 0.0001)

Research indicates a decrease in miR-214-3p levels across various cancers, including breast cancer, and it is being recognized as a crucial tumor suppressor [8, 9]. Our results confirmed a significantly reduced level of miR-214-3p in breast cancer tissues compared to adjacent non-cancerous tissues (Fig. 3A). Significantly, miR-214-3p mimics increased miR-214-3p levels in MCF-7 cells, while miR-214-3p inhibitor exhibited the opposite effect (Fig. 3B). Furthermore, overexpression of miR-214-3p notably led to an obvious decrease in cell viability and proliferation and significant enhancement of cell apoptosis in MCF-7 cells; however, downregulation of miR-214-3p exhibited the opposite effects (Fig. 3C and E). To sum up, miR-214-3p exerts tumor-suppressive effects in breast cancer.

### IKBKB is a direct target of miR-214-3p

**Fig. 4:**
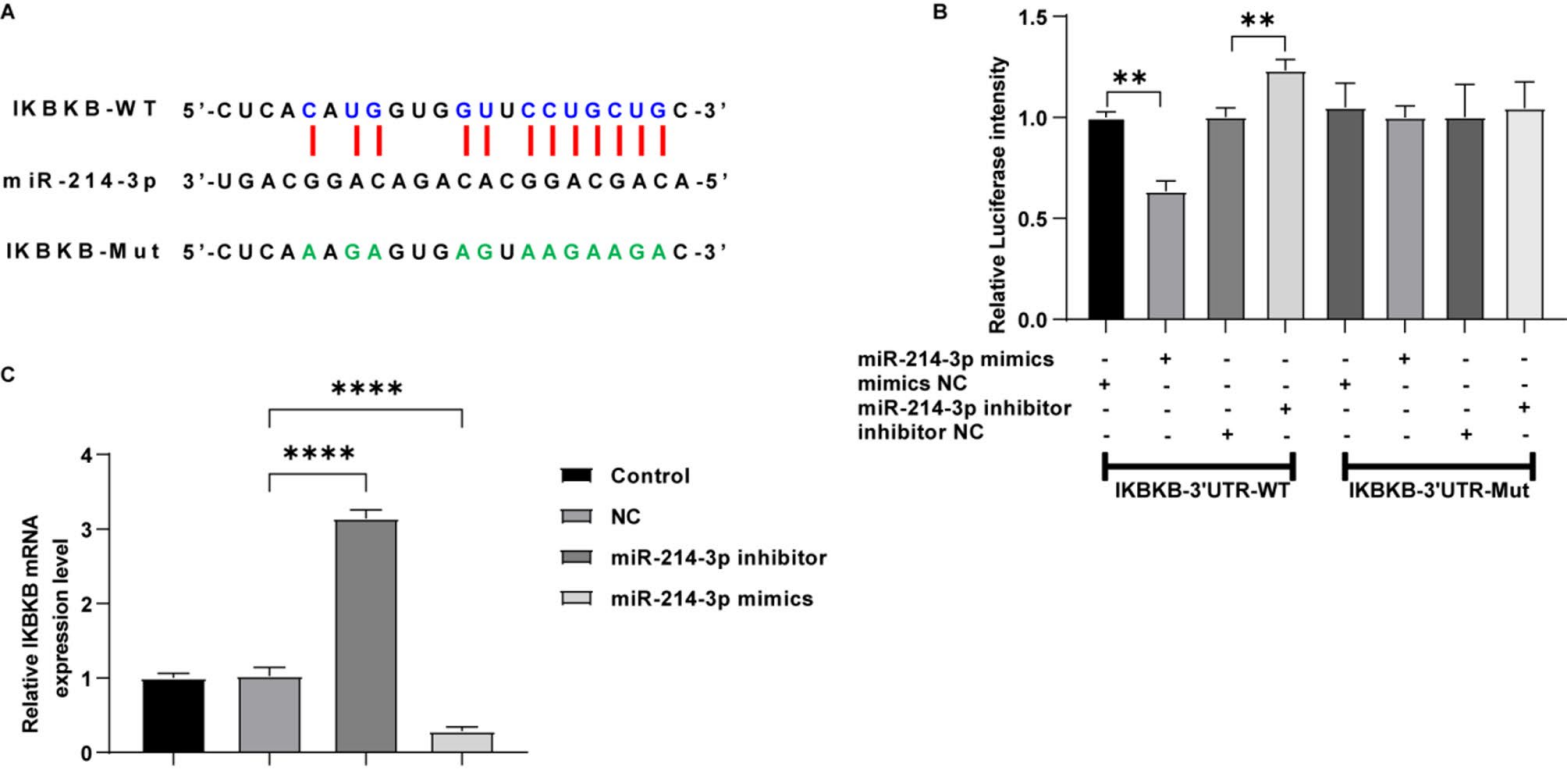
IKBKB is a target of miR-214-3p. (**A**) The predicted binding site between miR-214-3p and IKBKB. (**B**) The interaction between IKBKB and miR-214-3p was verified by dual-luciferase reporter assay (*n* = 3; two-way ANOVA; **P < 0.01). (**C**) RT-qPCR analysis of IKBKB levels in MCF-7 cells transfected with NC, miR-214-3p-mimics or miR-214-3p-inhibitor (*n* = 3; one-way ANOVA; ****P < 0.0001)

Next, we sought to identify the downstream targets of miR-214-3p. Through bioinformatics analysis, we screened potential target genes for miR-214-3p, and found that IKBKB may be the most likely target gene, which functions as tumor promoter in cancers [17, 18]. The binding sites between miR-214-3p and IKBKB were predicted using the STARBASE database (Fig. 4A). As shown in Fig. 4B, miR-214-3p overexpression reduced the luciferase activity of the wild type of IKBKB 3’UTR, while inhibition of its expression significantly increased the luciferase activity of the wild type of IKBKB 3’UTR (Fig. 4B). These results suggested that IKBKB is a direct target of miR-214-3p.

Furthermore, miR-214-3p inhibitor notably elevated the expression level of IKBKB in MCF-7 cells; whereas miR-214-3p mimics significantly reduced IKBKB levels in cells (Fig. 4C). These suggest that miR-214-3p negatively regulates the expression of IKBKB.

### CircRNA-14,052 affects the breast cancer progression in vitro via miR-214-3p/IKBKB axis

**Fig. 5:**
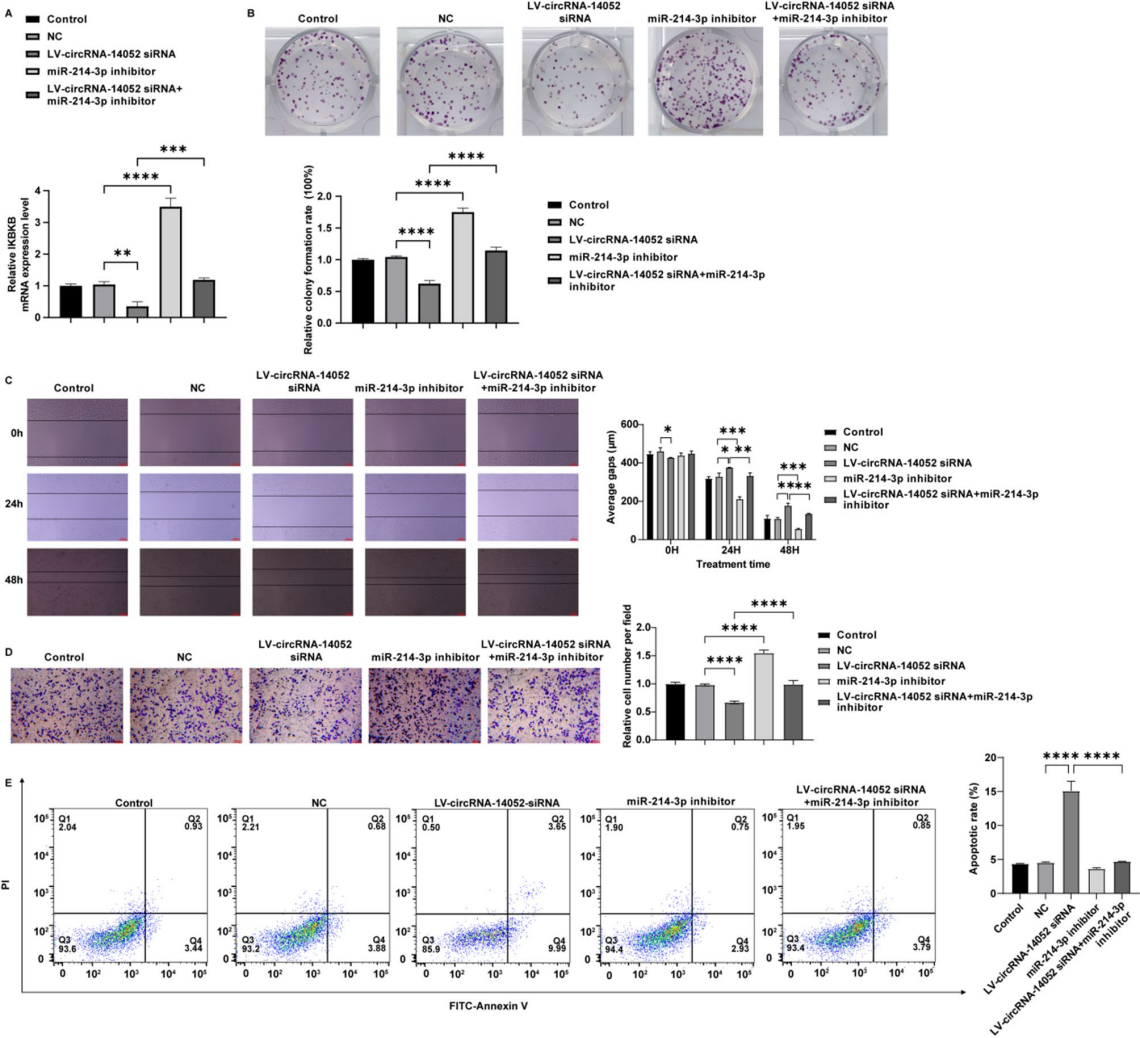
The role of circRNA-14,052/miR-214-3p axis in breast cancer. MCF-7 cells were transfected with LV-NC, LV-circRNA-14,052-siRNA, miR-214-3p-inhibitor, and LV-circRNA-14,052-siRNA + miR-214-3p-inhibitor. (**A**) The expression level of IKBKB in MCF-7 cells was detected by RT-qPCR (*n* = 3; one-way ANOVA; **P < 0.01, ***P < 0.001, ****P < 0.0001). (**B**) Cell proliferation was assessed by the colony formation assay (*n* = 3; one-way ANOVA; ****P < 0.0001). (**C**) Cell migration was determined by the wound-healing assay (*n* = 3; two-way ANOVA; *P < 0.05, **P < 0.01, ***P < 0.001). (**D**) The invasion of cancer cells was detected by transwell assay (*n* = 3; one-way ANOVA; ****P < 0.0001). (**E**) The apoptotic rate of the cancer cells was detected by flow cytometry (*n* = 3; one-way ANOVA; ****P < 0.0001)

Given the observed oncogenic effects of circRNA-14,052 and tumor-suppressive effects of miR-214-3p in breast cancer, we then performed rescue assays to investigate whether circRNA-14,052 promotes breast cancer progression by sponging miR-214-3p to regulate IKBKB expression. As shown in Fig. 5A, circRNA-14,052 deficiency significantly downregulated the expression of IKBKB in MCF-7 cells compared to the NC group; however, that change was reversed by miR-214-3p inhibitor. Additionally, circRNA-14,052 deficiency notably reduced MCF-7 cell proliferation, migration and invasion and increased cell apoptosis (Fig. 5B and E). However, inhibition of miR-214-3p remarkably reversed the inhibitory effects of circRNA-14,052 silence on MCF-7 cell growth (Fig. 5B and E). A similar phenomenon was noted in a different breast cancer cell line, MDA-MB-231 (Figure S1A-S1F). Our findings revealed that circRNA-14,052 deficiency significantly impaired the viability of MDA-MB-231 cells and downregulated IKBKB expression in MDA-MB-231 cells; in contrast, miR-214-3p knockdown exerted the opposing effects, markedly enhancing cell viability and upregulating IKBKB levels in MDA-MB-231 cells (Figure S1A-S1F). Importantly, the inhibitory effects of circRNA-14,052 deficiency on cell cell viability and IKBKB expression in MDA-MB-231 cells were notably reversed by miR-214-3p inhibition (Figure S1E-S1F). These findings underscore the critical role of the circRNA-14,052/miR-214-3p/IKBKB axis in the progression of breast cancer.

**Fig. 6:**
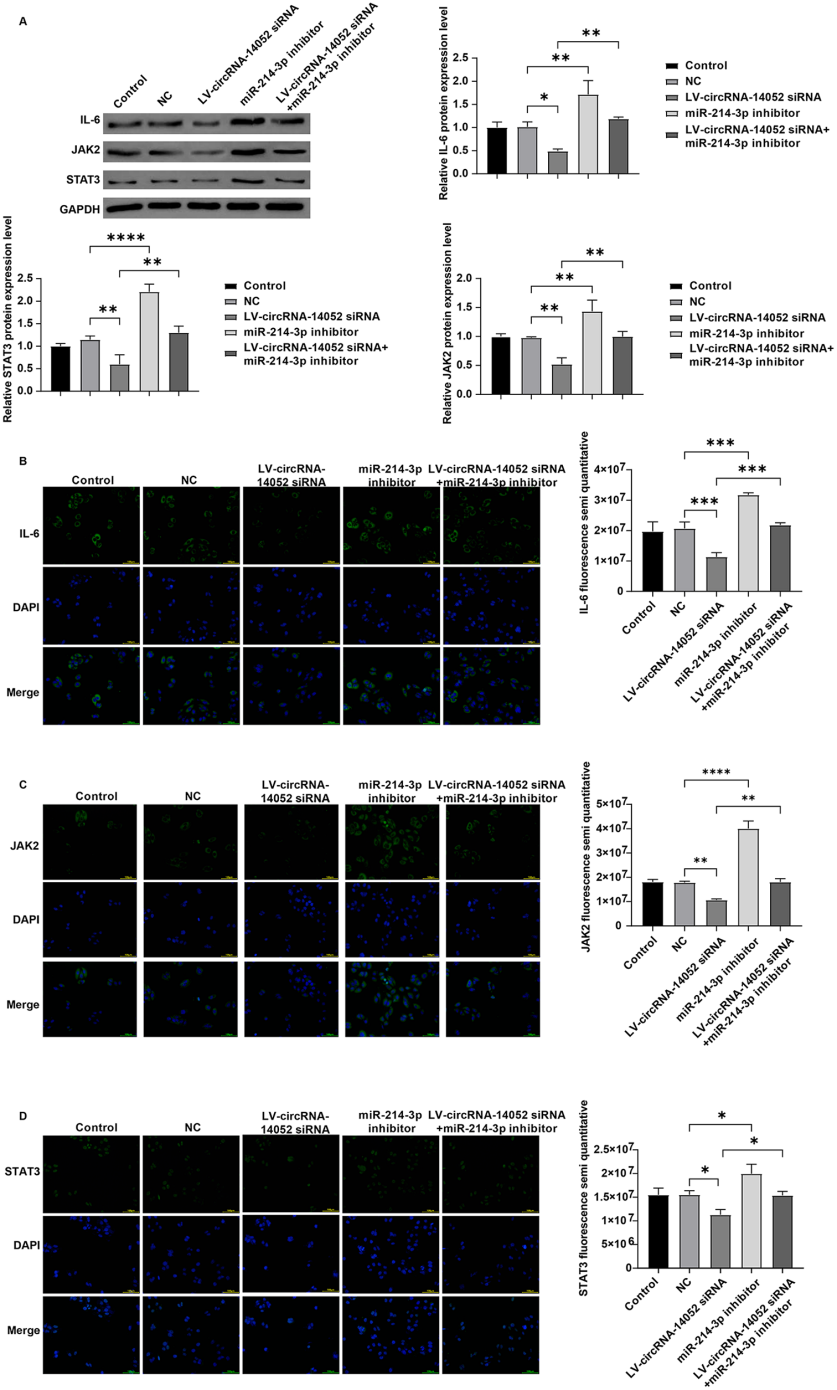
circRNA-14,052/miR-214-3p axis regulates the expression of IL-6, **JAK2**, **and STAT3.** MCF-7 cells were transfected with LV-NC, LV-circRNA-14,052-siRNA, miR-214-3p-inhibitor, and LV-circRNA-14,052-siRNA + miR-214-3p-inhibitor. (**A**, **B**, **C**, **D**) Western blot and IF assays were conducted to determine the expression levels of IL-6, JAK2, and STAT3 in MCF-7 cells (*n* = 3; one-way ANOVA; *P < 0.05, **P < 0.01, ***P < 0.001, ****P < 0.0001)

Furthermore, the knockdown of miR-214-3p increased the expression levels of IL6, JAK2, and STAT3 in MCF-7 cells (Fig. 6A and D). Conversely, these protein expressions was significantly downregulated in MCF-7 cells when circRNA-14,052 was silenced, but the co-silence of miR-214-3p reversed these changes (Fig. 6A and D). Collectively, circRNA-14,052 knockdown could inhibit the breast cancer progression in vitro via miR-214-3p/IKBKB/IL-6/JAK2/STAT3 axis.

### CircRNA-14,052 affects the breast cancer progression in vivo via miR-214-3p/IKBKB axis

**Fig. 7:**
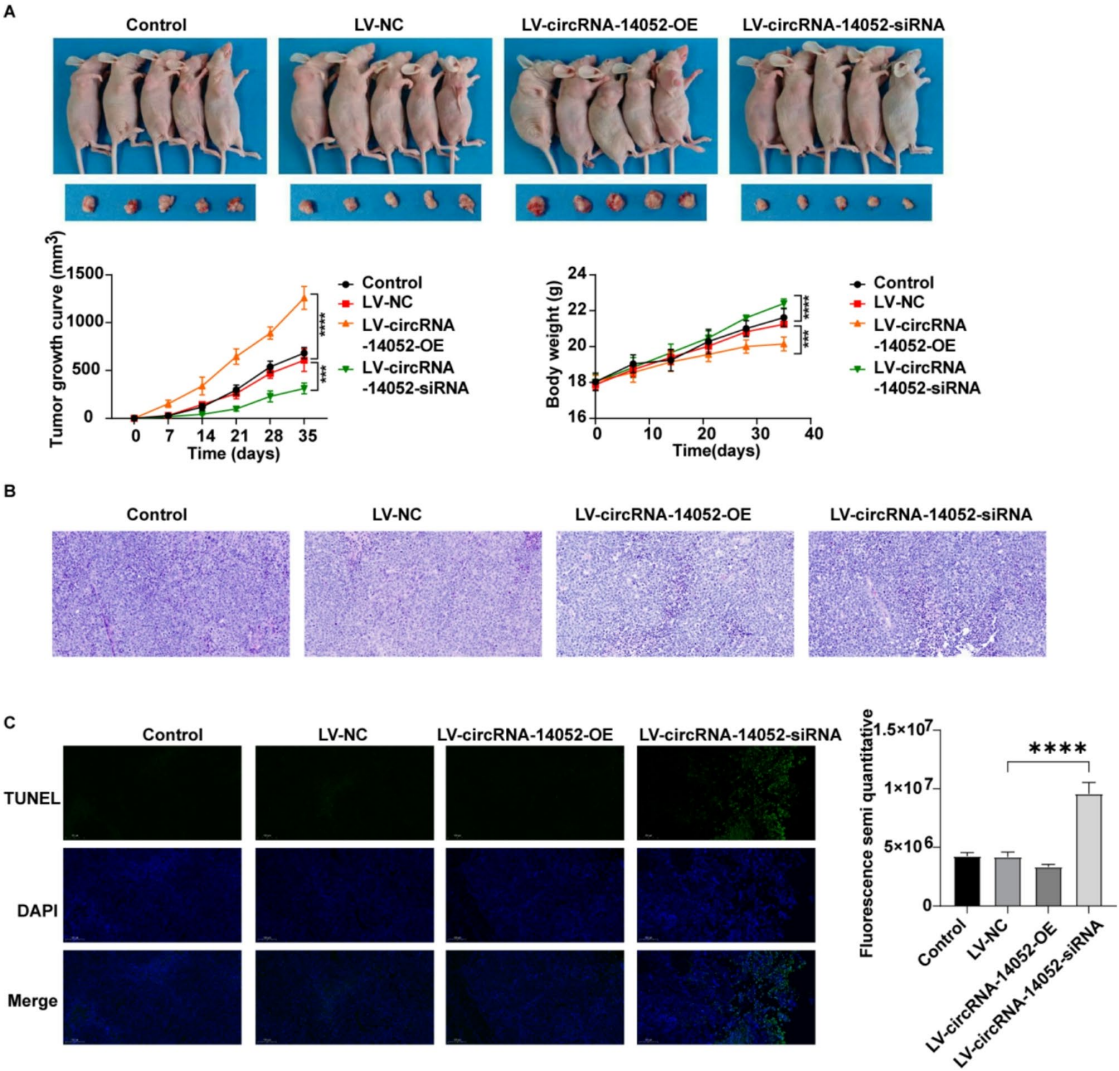
The role of the circRNA-14052 in breast cancer in vivo. (**A**) Photographs of tumors in the four groups on days 0, 7, 14, 21, 28, and 35. The size of tumors and body weight of the mice in each group were recorded on days 0, 7, 14, 21, 28, and 35 (*n* = 5; two-way ANOVA; ***P < 0.001, ****P < 0.0001). (**B**) H&E staining of the pathological tissues of mice. (**C**) Cell apoptosis was evaluated by TUNEL staining analysis (*n* = 3; one-way ANOVA; ****P < 0.0001). Blue color, nuclei stained by DAPI; green color, TUNEL positive cells

**Fig. 8:**
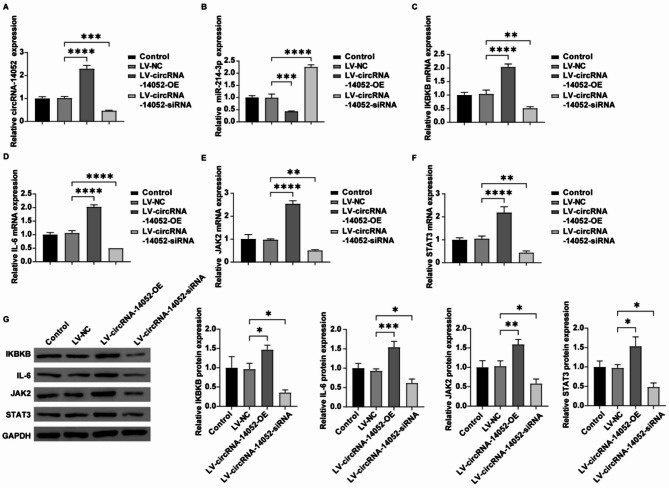
The circRNA-14,052 affects the breast cancer progression in vivo via miR-214-3p/IKBKB/IL6/JAK2/STAT3 axis. (**A**, **B**) The relative expression levels of circRNA-14,052 and miR-214-3p in tumor tissues from mice were detected by RT-qPCR (*n* = 3; one-way ANOVA; **P < 0.01, ***P < 0.001, ****P < 0.0001). (**C-G**) RT-qPCR and western blot were performed to determine the mRNA and protein levels of IKBKB, IL6, JAK2, and STAT3 in tumor tissues from mice (*n* = 3; one-way ANOVA; *P < 0.05, **P < 0.01, ***P < 0.001, ****P < 0.0001)

Based on our in vitro results indicating that the knockdown of circRNA-14,052 led to decreased proliferation, alongside an enhancement of apoptosis in MCF-7 cells, we next sought to validate these effects in vivo. Results showed that deficiency of circRNA-14,052 led to a significant decrease in the tumor size and an increase in the body weight of nude mice (Fig. 7A). In contrast, the opposite trend could be observed in the circRNA-14,052 overexpression group (Fig. 7A). Moreover, deficiency of circRNA-14,052 obviously enhanced cell apoptosis in tumor tissues from MCF-7 tumor-bearing mice compared to the LV-NC group (Fig. 7B, 7 C). Meanwhile, deficiency of circRNA-14,052 led to a significant decrease in circRNA-14,052, IKBKB, IL-6, JAK2, and STAT3 levels, and an increase in miR-214-3p levels in tumor tissues from MCF-7 tumor-bearing mice compared to the LV-NC group (Fig. 8A-G). Conversely, circRNA-14,052 overexpression exhibited the opposite effects (Fig. 8A-G). To sum up, circRNA-14,052 knockdown could inhibit the breast cancer progression in vivo via miR-214-3p/IKBKB axis.

## Discussion

In this study, we identified circRNA-14,052 as a novel oncogenic circRNA that promotes breast cancer progression by sponging miR-214-3p and upregulating IKBKB, thereby activating the IL-6/JAK2/STAT3 signaling axis. These results establish a previously unrecognized ceRNA regulatory network in breast cancer pathogenesis, highlighting circRNA-14,052 and its downstream effectors as potential therapeutic targets for breast cancer treatment.

Unlike linear RNA, circular RNA forms a closed loop and lacks both a 5’ cap and a 3’ poly (A) tail, which renders it less susceptible to degradation by RNA exonucleases [19]. It has been shown that circRNAs are participated in the development of various diseases, including neurological disorders, cardiovascular diseases, and tumors [20, 21]. Accumulating evidence has firmly established that dysregulated circRNAs serve as pivotal regulators in tumorigenesis and cancer progression across malignancies [22–24]. Wang et al. demonstrated that circ_0007823 drives TNBC progression and contributes to resistance against cisplatin through the miR-182-5p/FOXO1 axis [25]. Yang et al. revealed circRNA_100876 enhances tumor growth and metastasis by competitively binding miR-361-3p [26]. Liu et al. identified circRNA_103809 as a tumor suppressor that inhibits breast cancer progression via targeting miR-532-3p [27]. These previous studies have shown that abnormal expression of circRNAs could regulate the progression of breast cancer by affecting the proliferation, apoptosis, and drug resistance of cancer cells. Thus, circRNAs can be a potential biomarker or therapeutic target for breast cancer. In this study, we found that circRNA-14,052 levels were notably increased in breast cancer tissues versus adjacent non-cancerous tissues. Additionally, overexpression of circRNA-14,052 significantly enhanced MCF-7 cell proliferation, and reduced cell apoptosis; whereas, circRNA-14,052 deficiency exhibited the opposite effects (Fig. 1). These results demonstrate that circRNA-14,052 could act as a novel oncogenic driver in breast cancer pathogenesis.

It has been found that circular RNAs can competitively bind to miRNAs through complementary base pairing thereby relieving the inhibitory effect of miRNAs on target genes, which is known as the circRNA/miRNA regulatory axis [28, 29]. We found that miR-214-3p may be a potential binding target of circRNA-14,052. When circRNA-14,052 was silenced in MCF-7 cells, the miR-214-3p expression significantly increased; when circRNA-14,052 was overexpressed, the expression of miR-214-3p significantly decreased, indicating that circRNA-14,052 has a negative regulatory effect on miR-214-3p. MiR-214-3p, located on 1q24.3, is a single-stranded RNA with a length of 22 bases. This miRNA is frequently down-regulated in various cancers such as endometrial cancer, cervical cancer, and colorectal cancer, demonstrating tumor-suppressive effects [30–34]. Our results showed that overexpression of miR-214-3p notably suppressed MCF-7 cell proliferation and apoptosis, thereby confirming its role as a tumor suppressor in breast cancer. In agreement with our results, Han et al. reported that the up-regulation of miR-214-3p inhibits breast cancer cell proliferation [35], which supports our findings. However, research by Prabhakar et al. revealed that miR-214-3p can also exert an oncogenic role in melanoma by targeting and suppressing two key negative regulators (ANKRD6 or CTBP1) of Wnt signaling, thereby indirectly activating β-catenin pathway [36]. These findings highlight the biological effects of miR-214-3p may be determined by its downstream target genes and cellular context. The function of miR-214-3p in tumors is complex. Thus, the dual roles of miR-214-3p in tumorigenesis warrant further systematic investigation to delineate its precise regulatory mechanisms and pathway in tumor cells.

In this study, we verified that IKBKB is a direct target gene of miR-214-3p through dual-luciferase assays and RT-qPCR (Fig. 4B and C). IKKβ, encoded by IKBKB gene, is a vital component of cytokine signaling activity as a subunit of IKK. Activated IKKβ phosphorylates NF-κB to dissociate and enter the nucleus to activate downstream genes, promoting tumor growth [37, 38]. Additionally, evidence has shown that IL-6 is a downstream gene of NF-κB, the activation of NF-κB leads to the activation of IL-6 and further initiates downstream signals [39, 40]. As it has been known that the aberrant activation of the JAK-STAT signaling pathway leads to the migration of tumor cells, several studies have shown that the JAK-STAT signaling pathway can intersect with other signaling pathways like NF-κB pathway and influence one another [41]. STAT3 is the downstream target JAK2, and both have been proved to be activated by the expression level of IL-6 [42]. The hyperactivated IL6/JAK/STAT3 can facilitate tumor cell proliferation and invasiveness; hence treatments targeting the IL6/JAK/STAT3 pathway are expected to inhibit tumor cell growth [43]. These results suggested that NF-κB signaling may be participated in cancer progression through IL6/JAK/STAT3 signaling. Our results showed that inhibition of miR-214-3p notably elevated IKBKB, IL6, JAK2, and STAT3 levels in MCF-7 cells (Fig. 6A and D). This suggests that the deficiency of miR-214-3p mediates the upregulation of IKBKB, which subsequently activates IL6/JAK/STAT3 signaling, thereby promoting tumor progression. Notably, our rescue experiments (Figs. 5 and 6) demonstrated that circRNA-14,052 knockdown led to a downregulation of IKBKB, IL-6, JAK2, and STAT3 levels in MCF-7 cells; however, these effects were reversed by miR-214-3p inhibition, confirming functional dependence on the miR-214-3p/IKBKB axis. These results demonstrated that circRNA-14,052 may promote the development of breast cancer by sponging miR-214-3p and regulating IKBKB/IL6/JAK/STAT3 signaling.

This study has several limitations. First, we investigated the role of the circRNA14052/miR-214-3p/IKBKB axis in only two breast cancer cell lines: MCF-7 (Luminal A cell line) and MDA-MB-231 (TNBC cell line). Future studies should expand to additional breast cancer cell lines (e.g., Luminal B and HER2-enriched breast cancer cell lines) to validate the biological functions and clinical relevance of circRNA14052 in breast cancer. Second, the regulatory effects of this axis in other cancer types remain unknown; thus, further research using diverse tumor models is needed to determine its universal role in cancer progression. Notably, liquid biopsy has emerged as a promising non-invasive tool for cancer detection and monitoring [44]. Recent studies highlight the potential of exosome- and platelet-derived circRNAs as biomarkers for liquid biopsy [45, 46]. Therefore, future work should explore whether circRNA14052 is detectable in circulation (e.g., plasma and exosomes) and evaluate its utility in liquid biopsy-based approaches for the early diagnosis and therapeutic monitoring of breast cancer. Additionally, the clinical specimens used for circRNA-14,052 expression analysis, though statistically significant, was relatively small. Future studies with expanded patient cohorts, including diverse subtypes and stages of breast cancer, will be necessary to validate our observations and further establish the diagnostic potential of circRNA-14,052.

## Conclusion

In conclusion, we found that circRNA-14,052 could promote the progression of breast cancer via the miR-214-3p/IKBKB/IL6/JAK/STAT3 axis. Thus, targeting circRNA14052/miR-214-3p/IKBKB axis may be a promising approach for the treatment of breast cancer. We hope our work may provide a new perspective on the treatment of breast cancer.

## Supplementary Information

Below is the link to the electronic supplementary material.

Supplementary Material 1

Supplementary Material 2

Supplementary Material 3

Supplementary Material 4

## Data Availability

No datasets were generated or analysed during the current study.
