# Supplementary material for: CircRNA-14052 promotes breast cancer progression via miR-214-3p/IKBKB pathway
Source: Hereditas. 2025 Oct 3;162:202. doi: 10.1186/s41065-025-00566-6 (PMC12495841; doi:10.1186/s41065-025-00566-6)
Supplement: Supplementary file 1 — Supplementary Material 1 [file 41065_2025_566_MOESM1_ESM.docx]

Table S1 Patient clinical information.

| **No.** | **Age** | **Tumor size** | **pTNM stage** | **lymph node** | **Histological grading** | **ER** | **PR** | **Her-2** | **Ki-67** | **E-cad** | **CK5/6** |
| --- | --- | --- | --- | --- | --- | --- | --- | --- | --- | --- | --- |
| 1 | 64 | 4.5*3.5*1.2cm | pT2N1M0 | (1/12) | Invasive carcinoma grade II, 7 score | 3+70% | 60%，3+ | 1+ | +10% | + | - |
| 2 | 59 | 1.8*1.6*1.5cm | pT1N0M0 | (0/8) | Invasive carcinoma grade II, 6 score | 3+80% | 20%，2+ | 1+ | +10% | + | - |
| 3 | 40 | 3*2*2cm | pT2N1M0 | (1/15) | Invasive carcinoma grade II, 7 score | 30%1+ | 15%，1+ | 2+ | 50% | - | - |
| 4 | 63 | 4*3*1cm | pT2N2M0 | (4/15) | Invasive carcinoma grade II, 7 score | 15%，1+ | - | 1+ | 30% | + | + |
| 5 | 53 | 5.5*2*1cm | pT3N2M0 | (5/24) | Invasive carcinoma grade II, 6 score | 90%，3+ | 90%，3+ | 0 | 5-8% | + | - |
| 6 | 69 | 2.2*2*2cm | pT2N0M0 | (0/15) | Invasive carcinoma grade II, 7 score | 70%，2+ | 60%，2+ | 1+ | 15% | + | - |
| 7 | 61 | 2*2*2cm | pT2N0M0 | (0/6) | Invasive carcinoma grade II, 6 score | 90%，3+ | 90%，3+ | 0 | 35%+ | - | - |
| 8 | 38 | 3*2*1cm | pT2N3M0 | (24/34) | Invasive carcinoma grade III, 6 score | 90%，3+ | 90%，3+ | 0 | 5-8% | - | - |
| 9 | 55 | 2*2*1cm | pT2N2M0 | (6/19) | Invasive carcinoma grade II, 7 score | 80%，2+ | 2%，+ | 0 | 3%+ | - | - |
