# Supplementary figures and images for: CircRNA-14052 promotes breast cancer progression via miR-214-3p/IKBKB pathway
Source: Hereditas. 2025 Oct 3;162:202. doi: 10.1186/s41065-025-00566-6 (PMC12495841; doi:10.1186/s41065-025-00566-6)

GAPDH-1

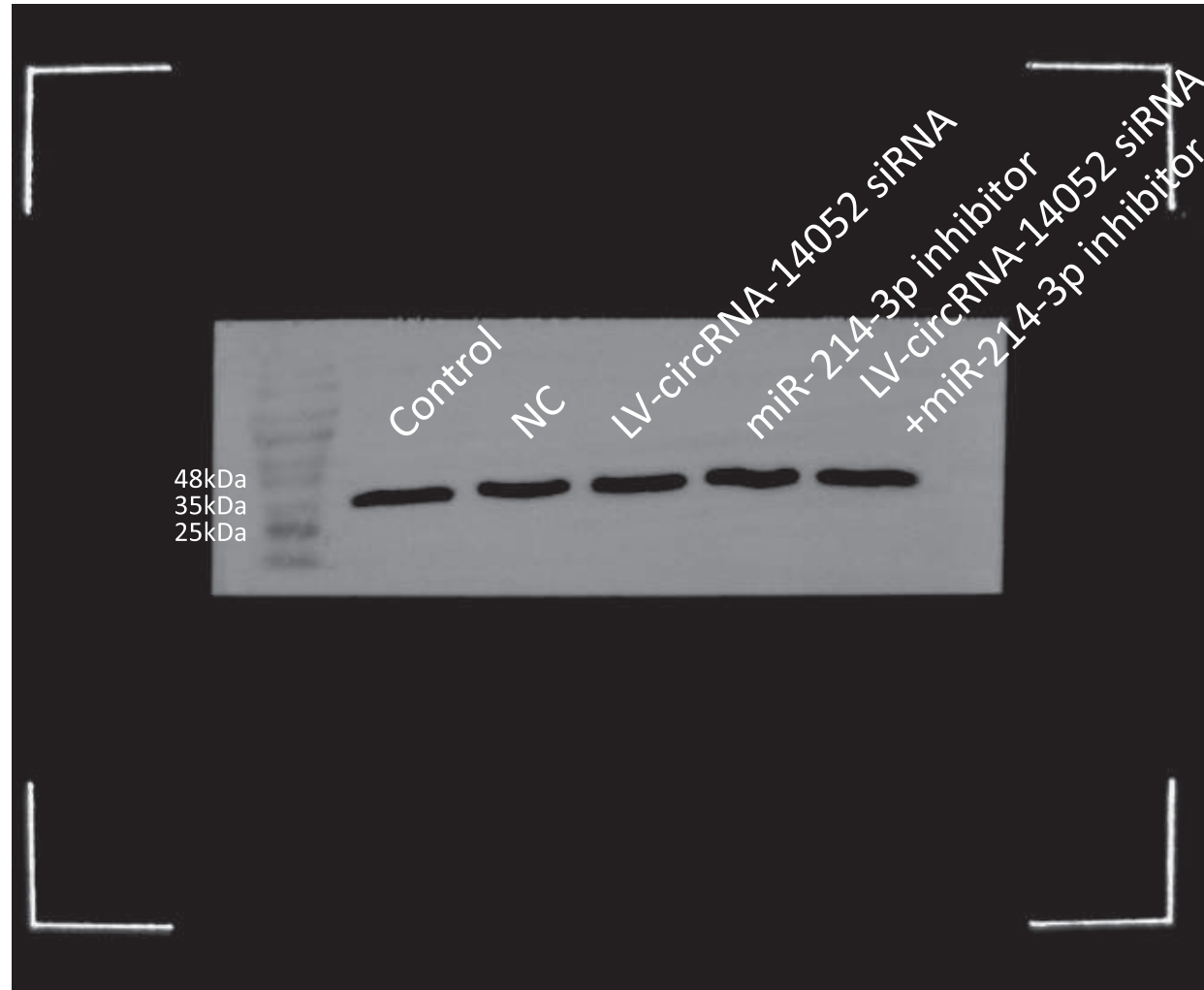

GAPDH-2

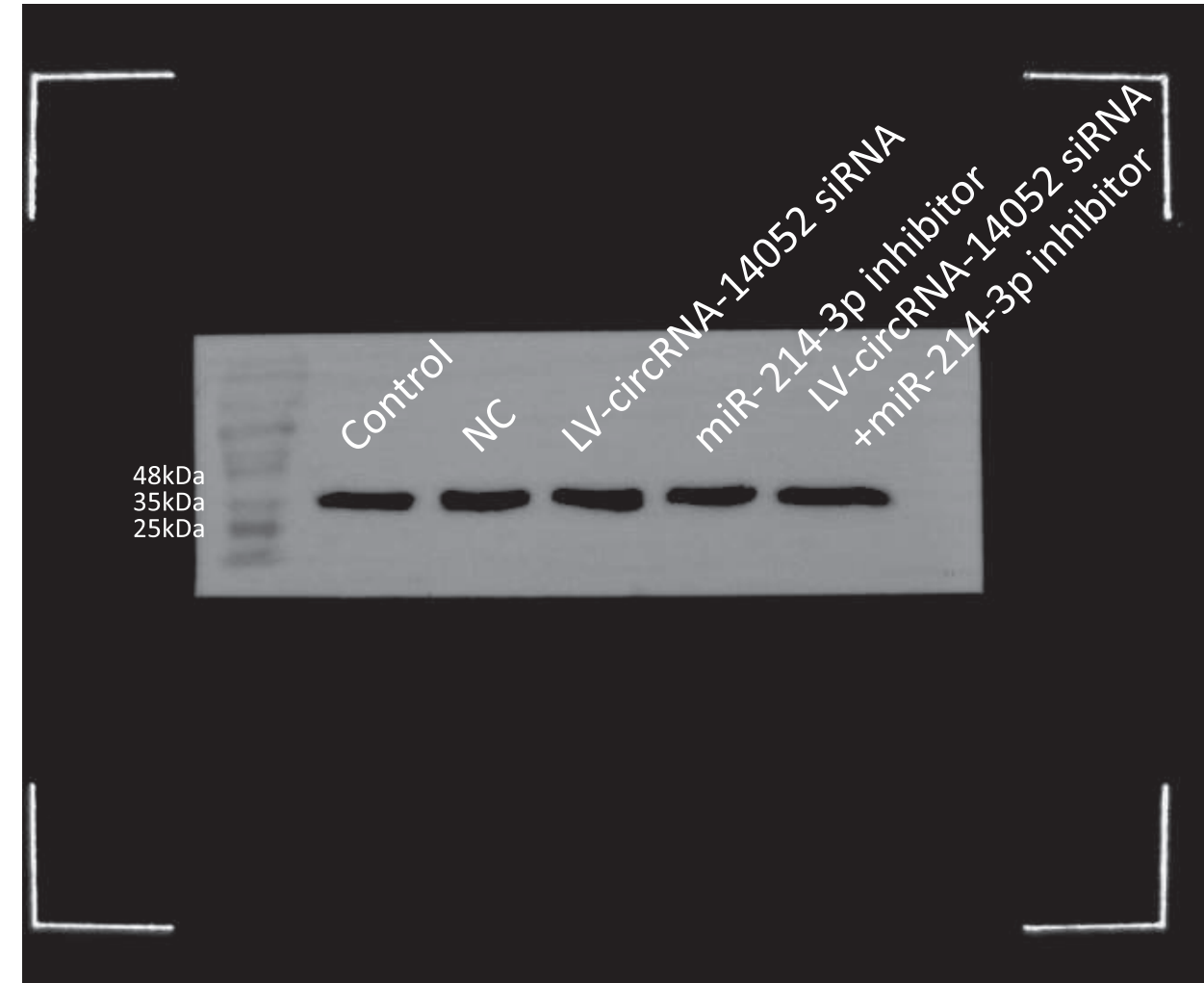

GAPDH-3

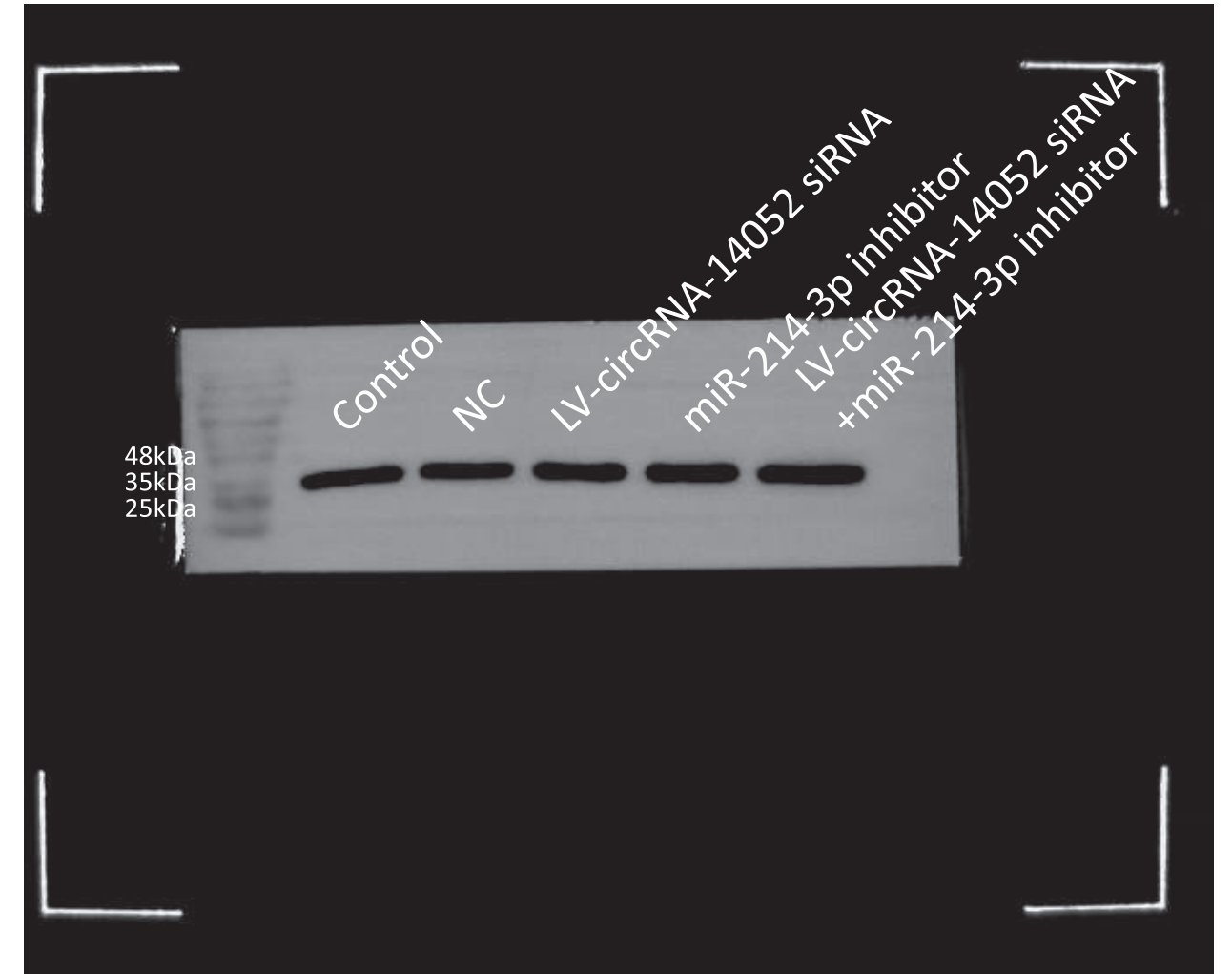

IL-6-1

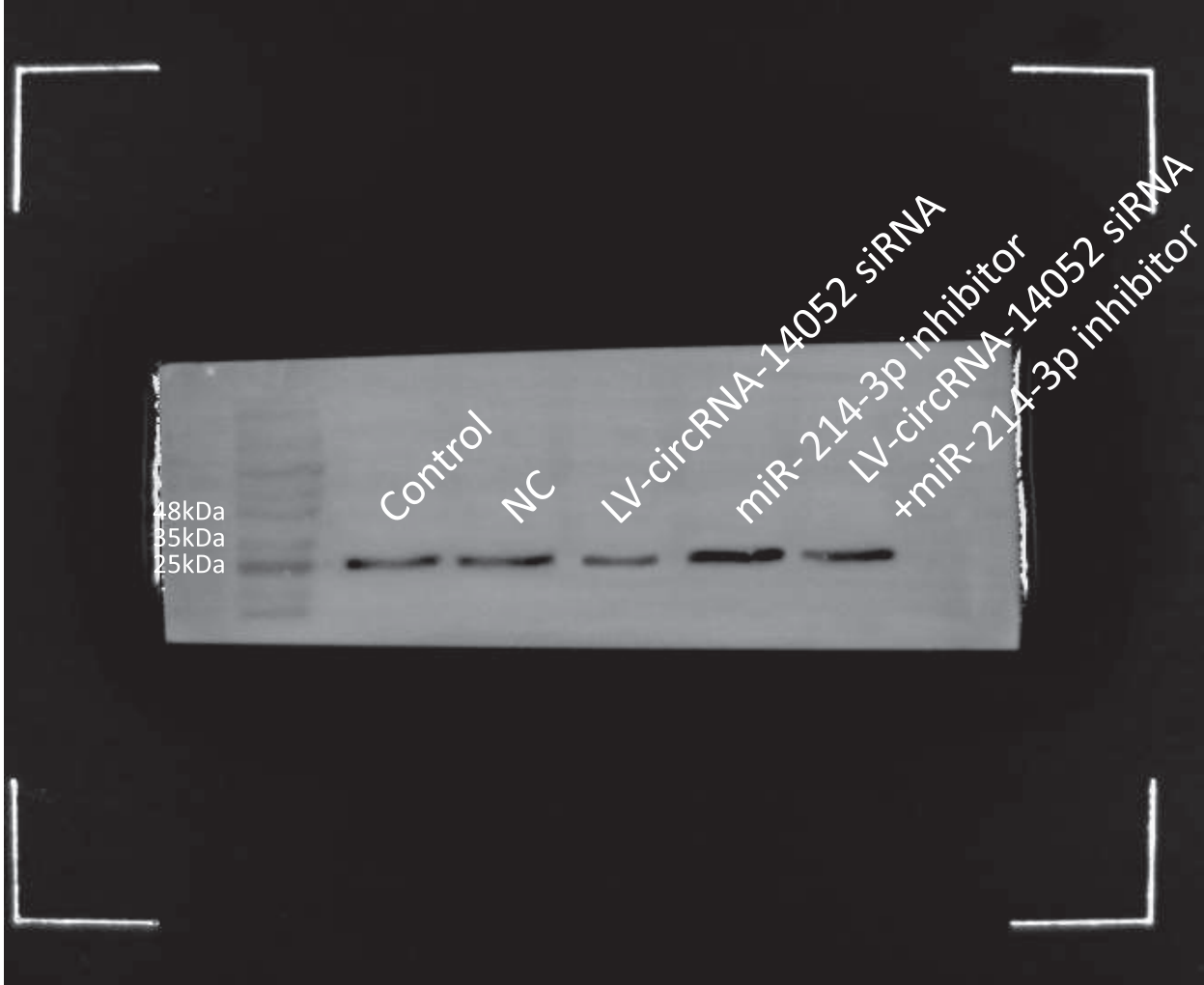

IL-6-2

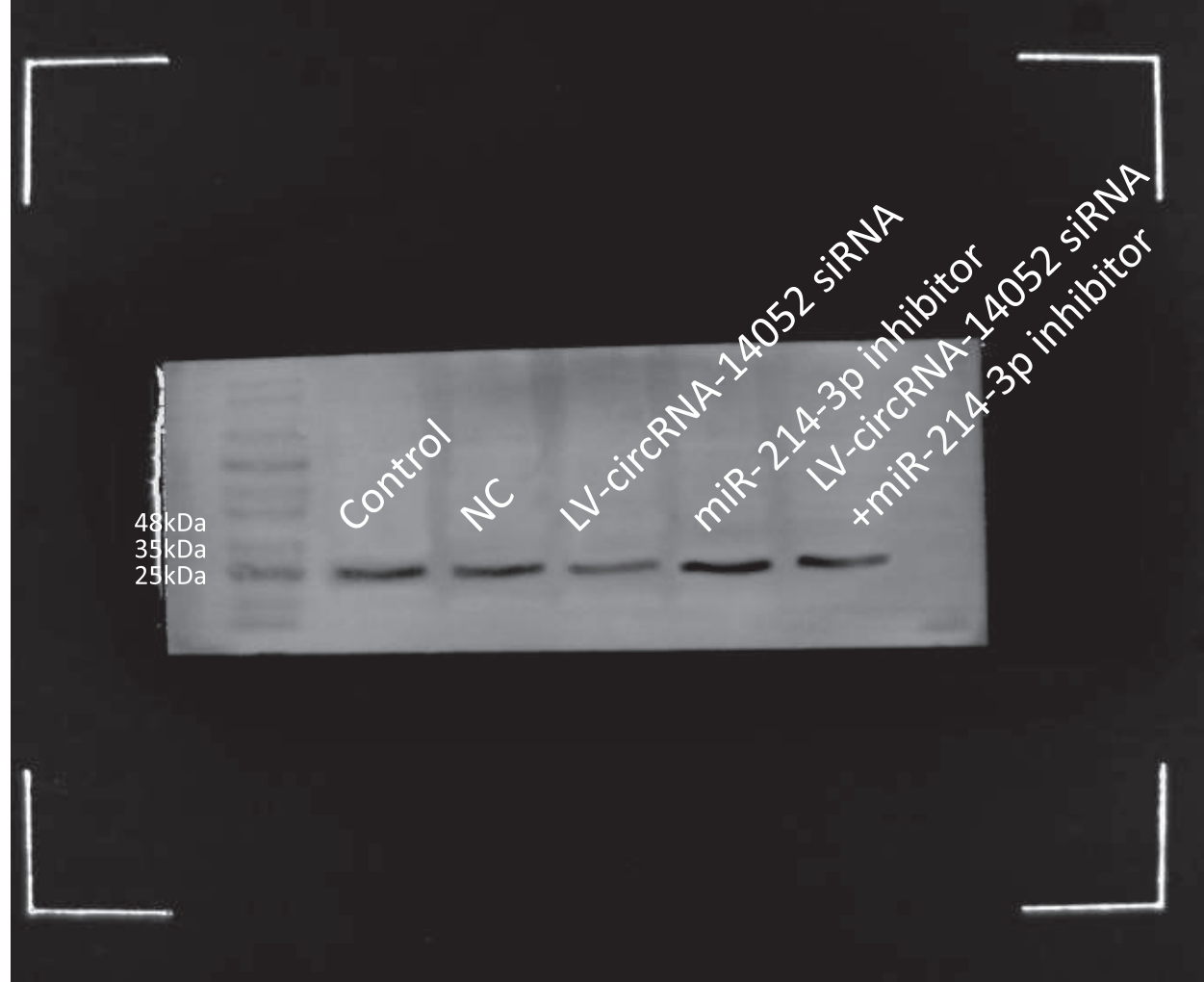

IL-6-3

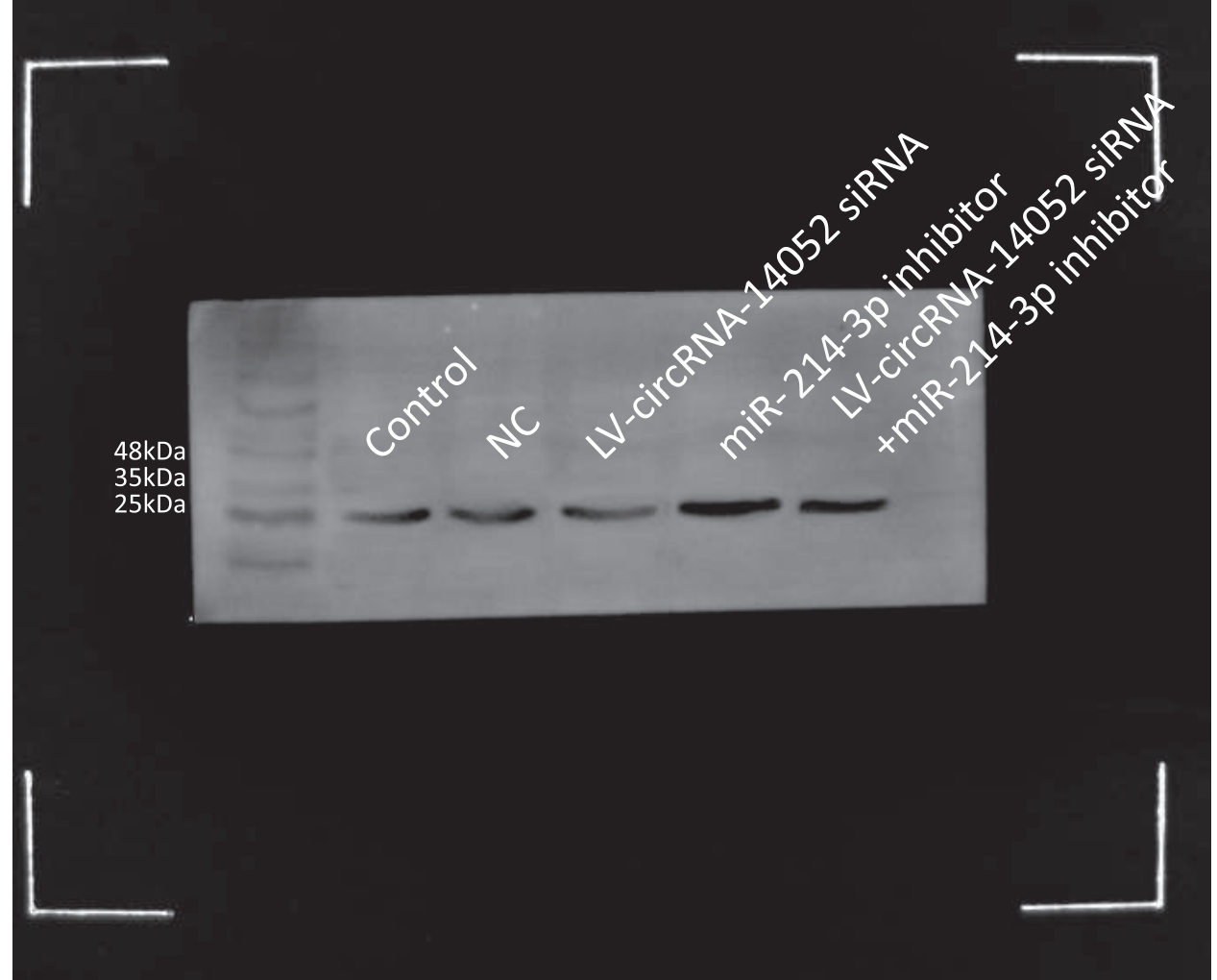

JAK2-1

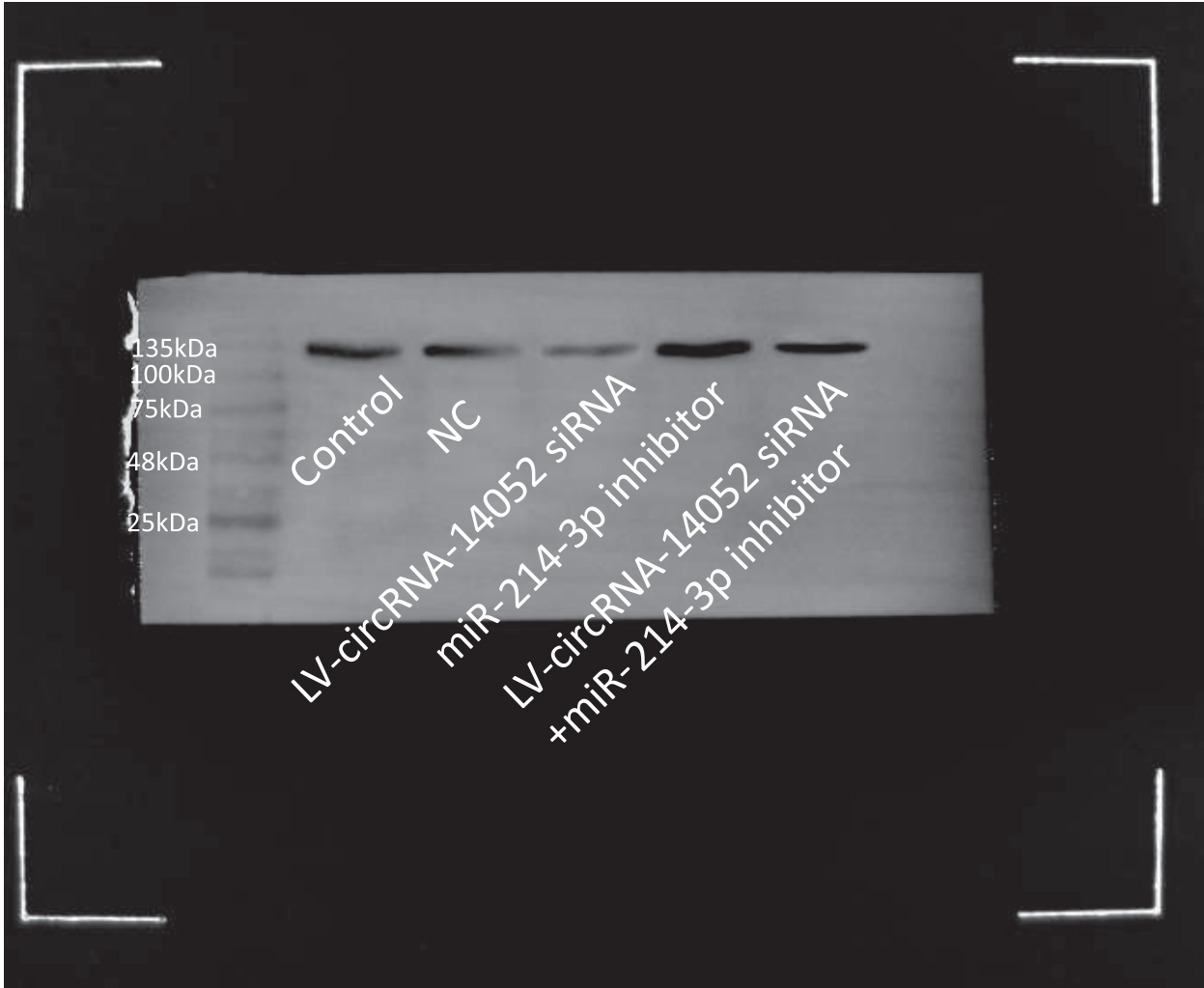

JAK2-2

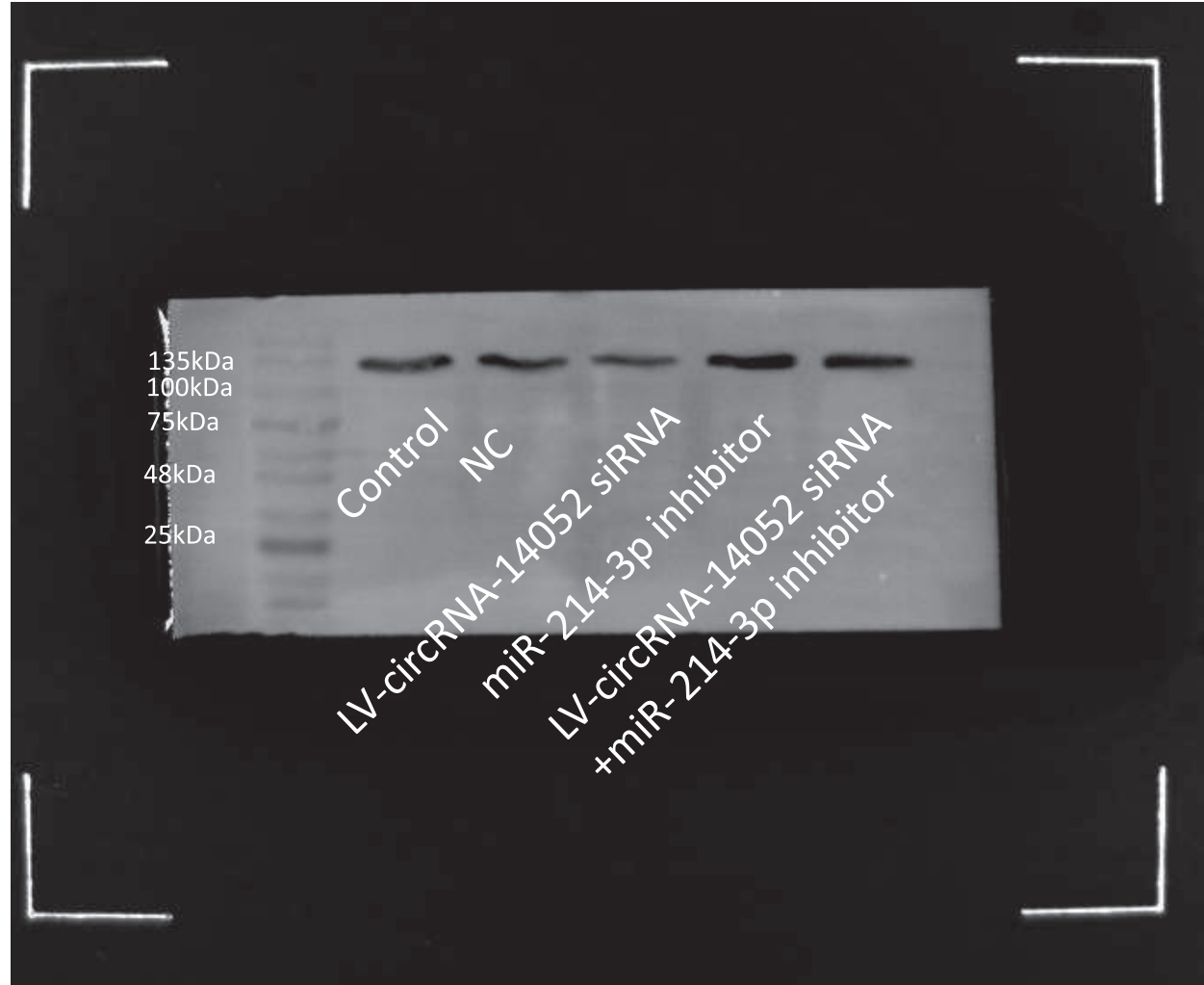

JAK2-3

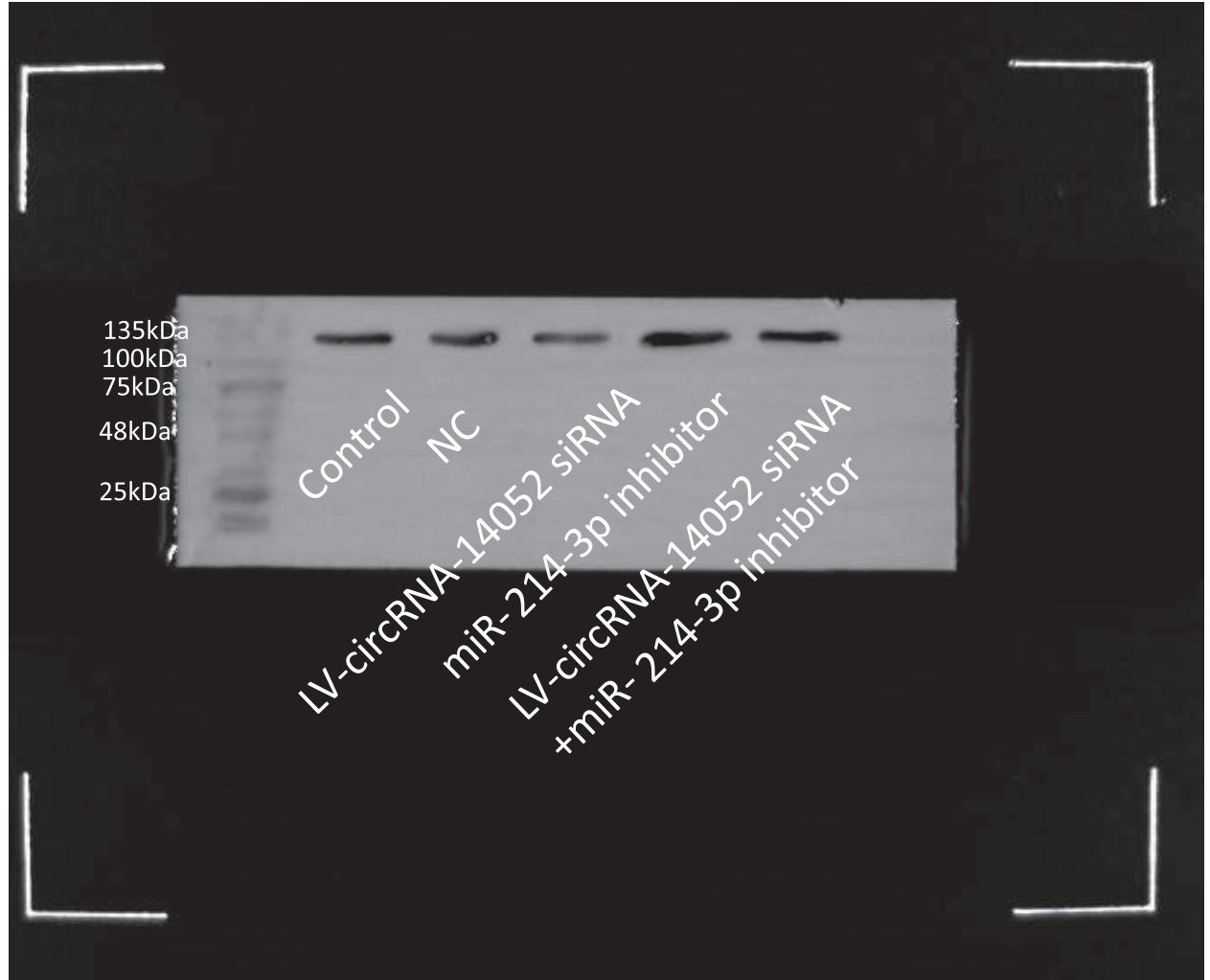

STAT3-1

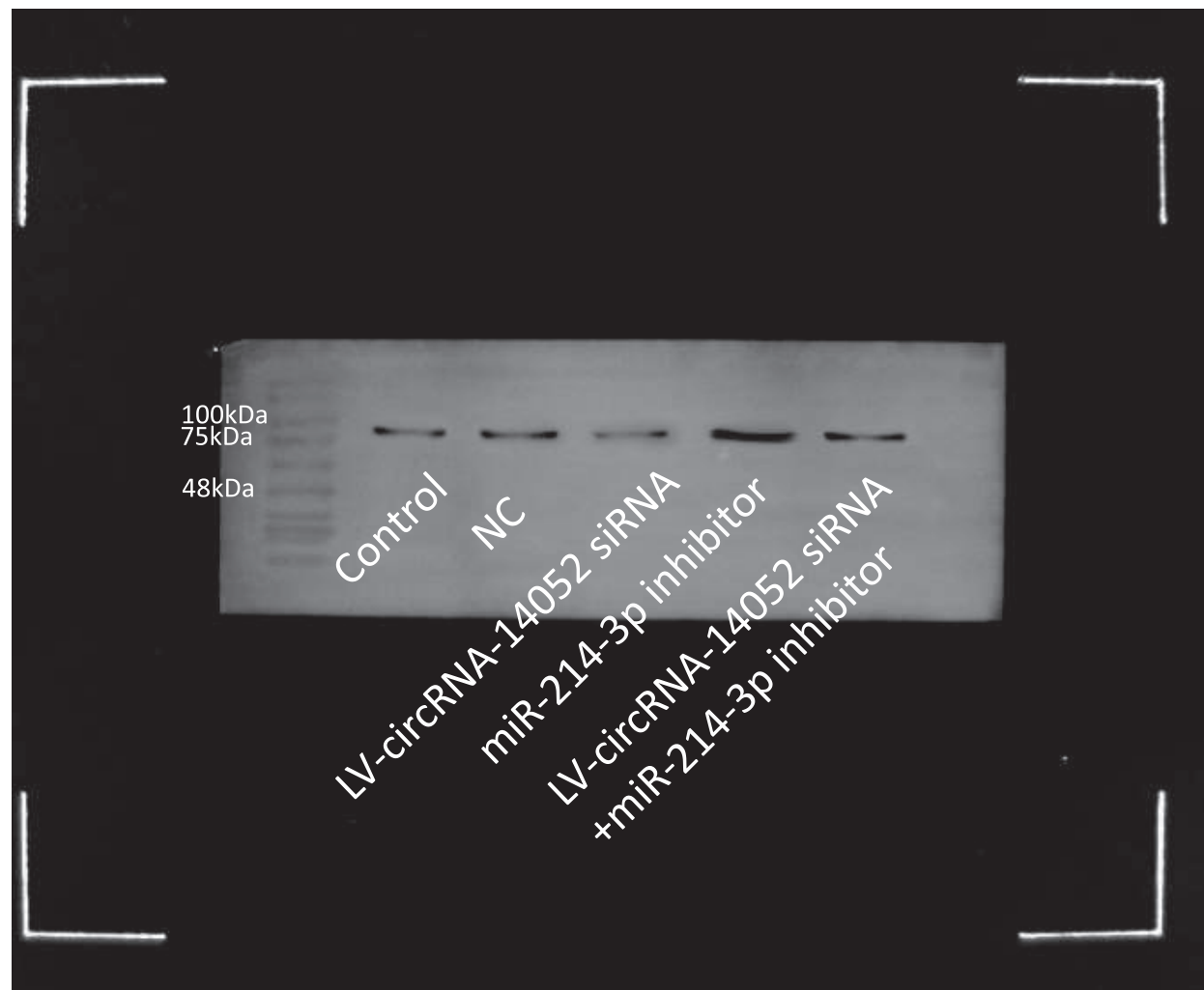

STAT3-2

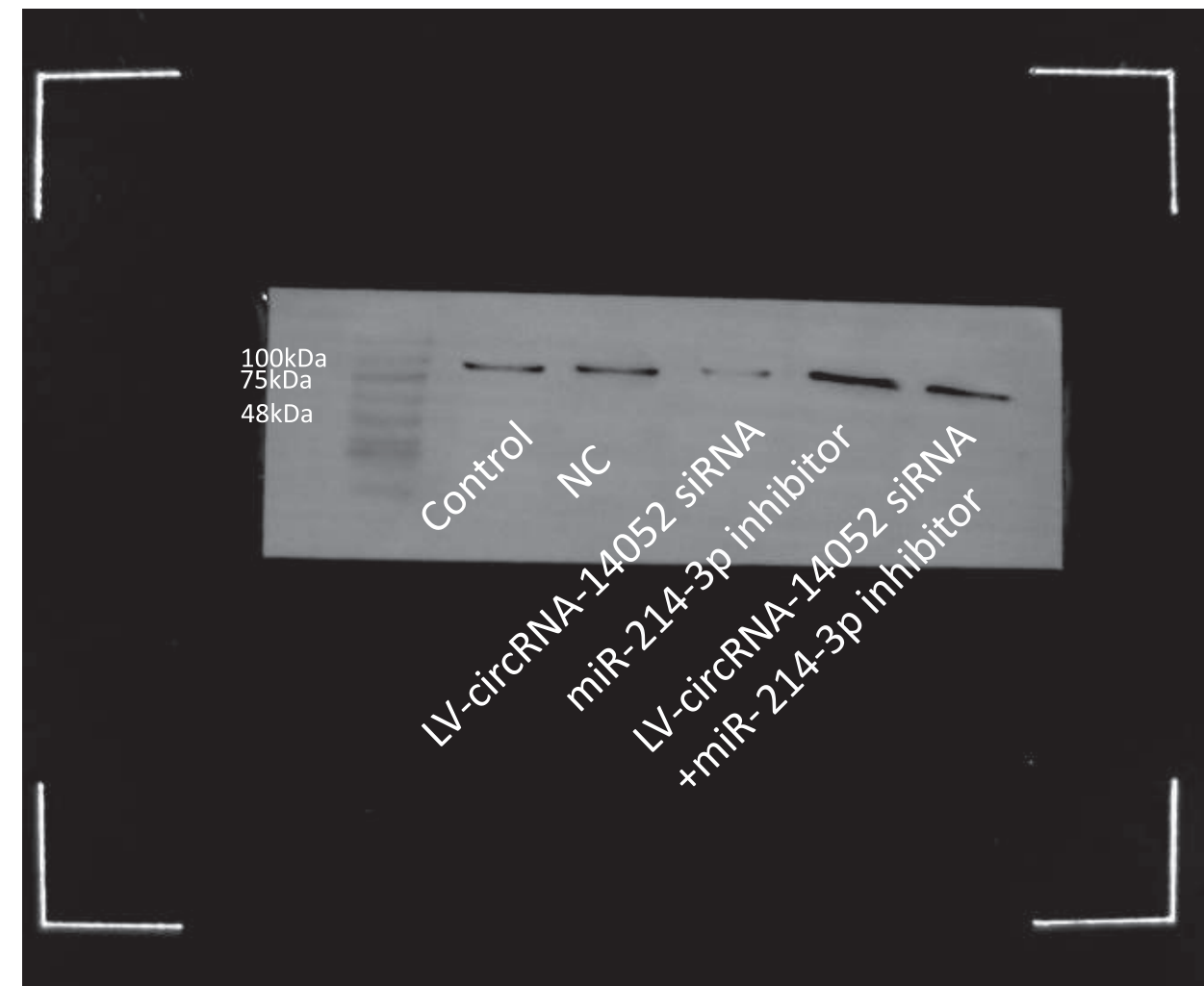

STAT3-3

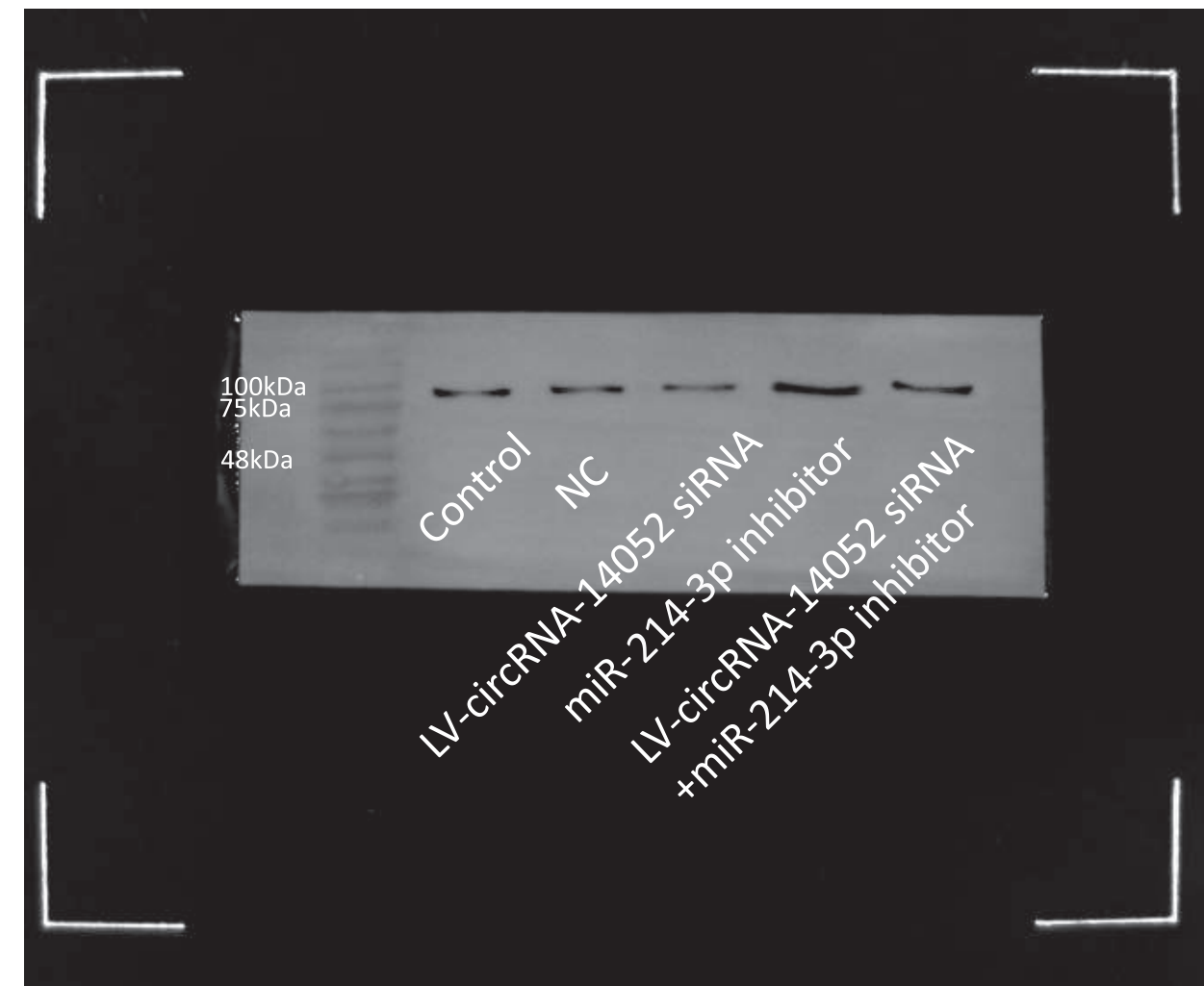

Supplement: Supplementary file 2 — Supplementary Material 2 [file 41065_2025_566_MOESM2_ESM.pdf]

GAPDH-1

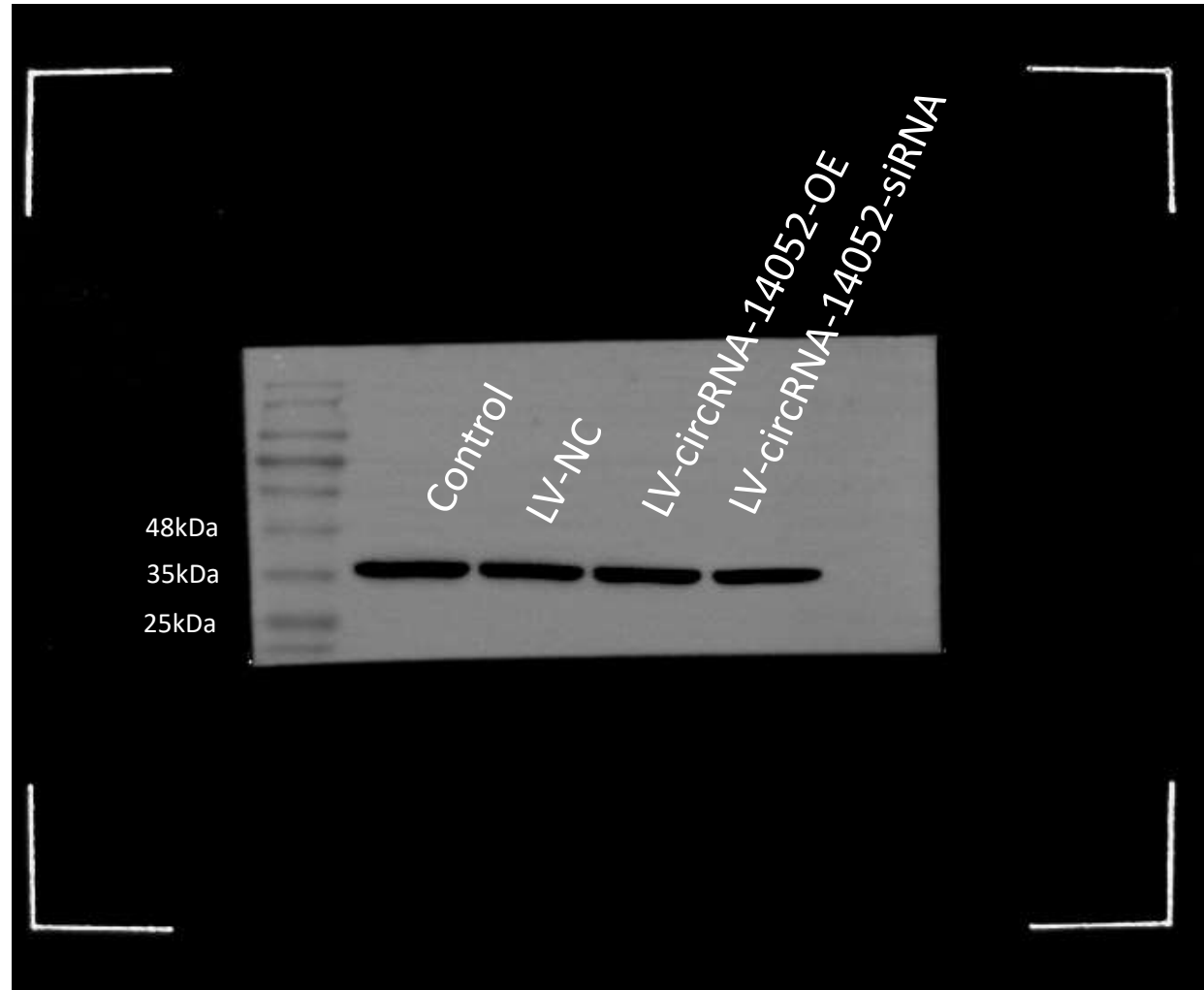

GAPDH-2

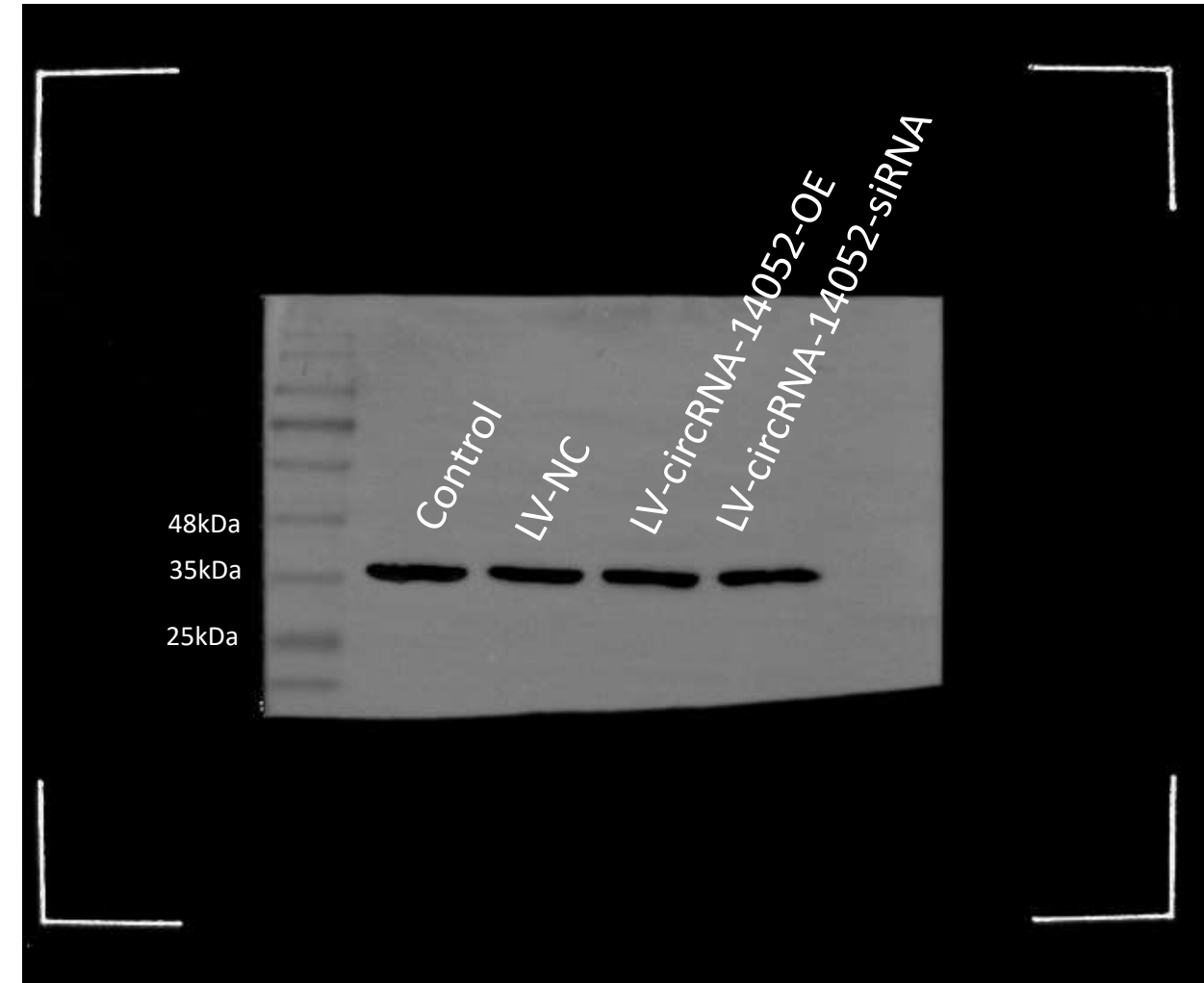

GAPDH-3

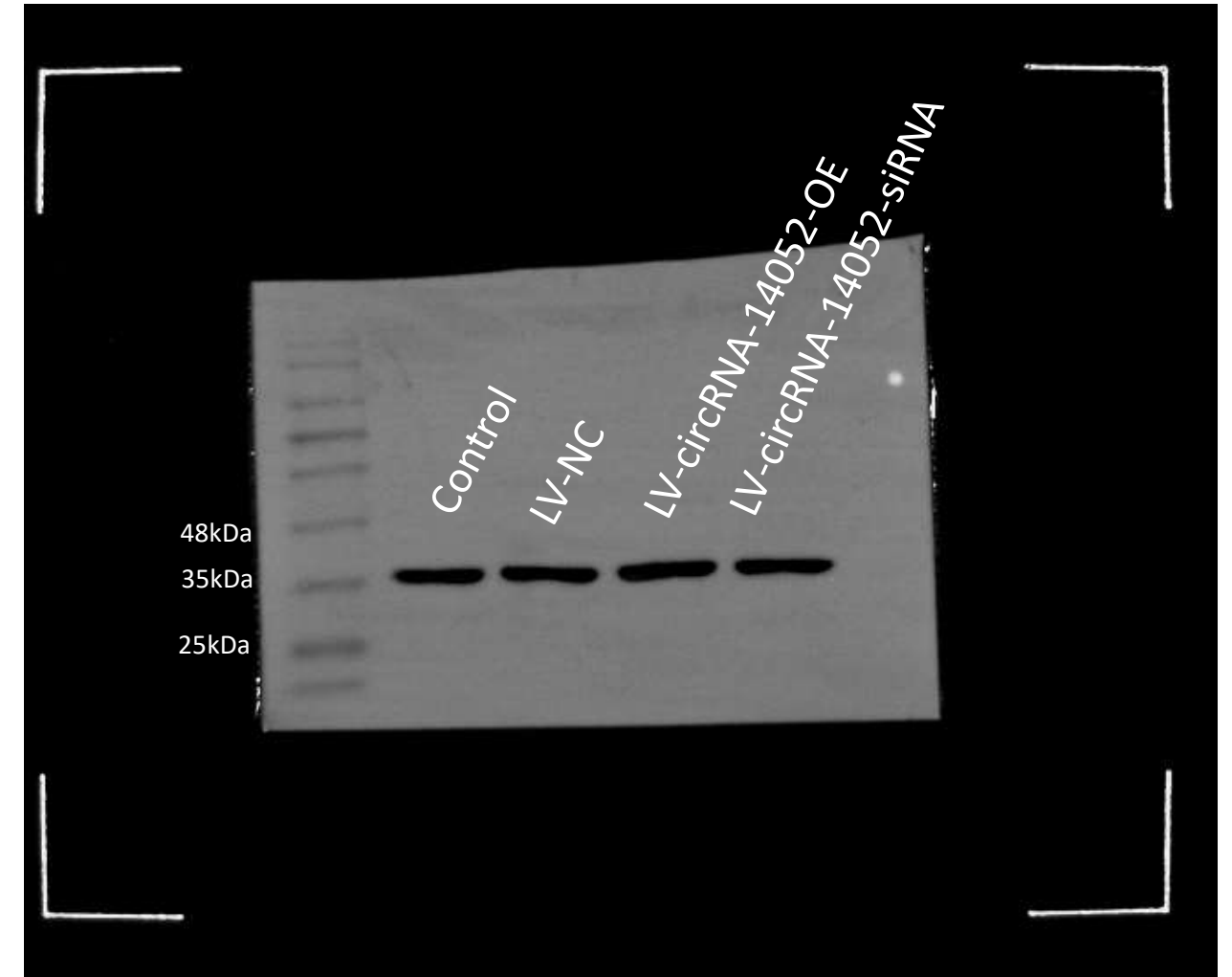

IKBKB-1

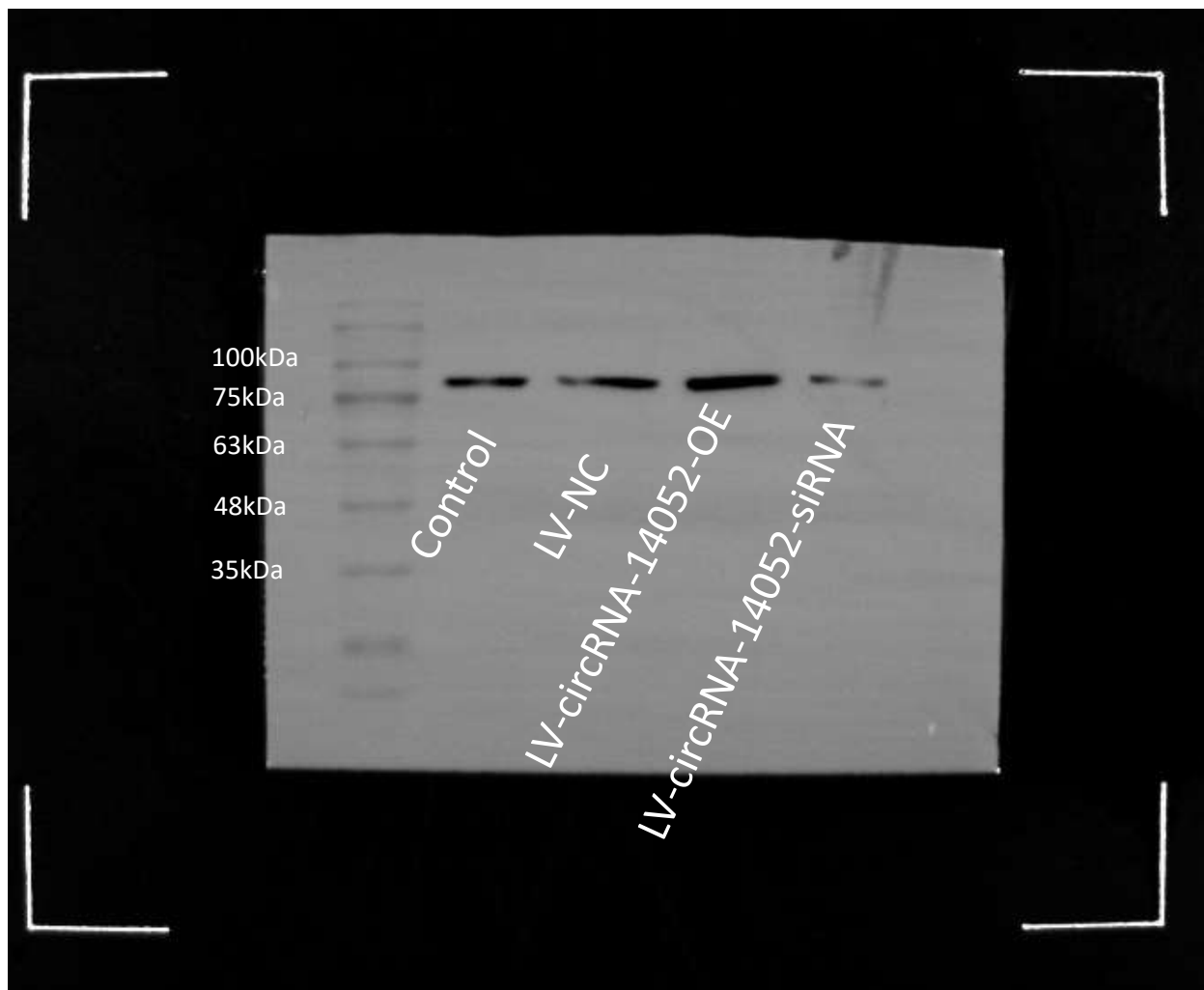

IKBKB-2

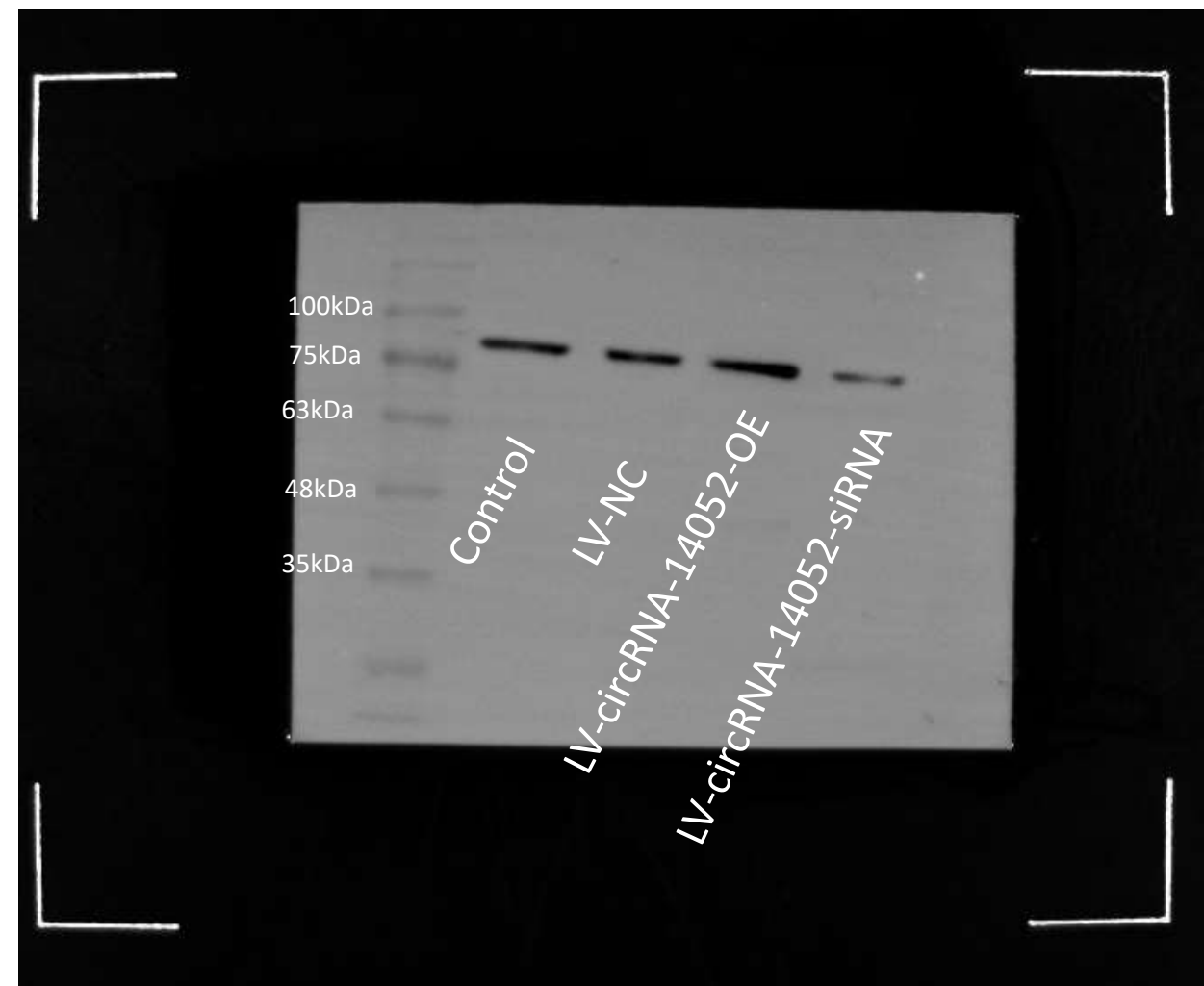

IKBKB-3

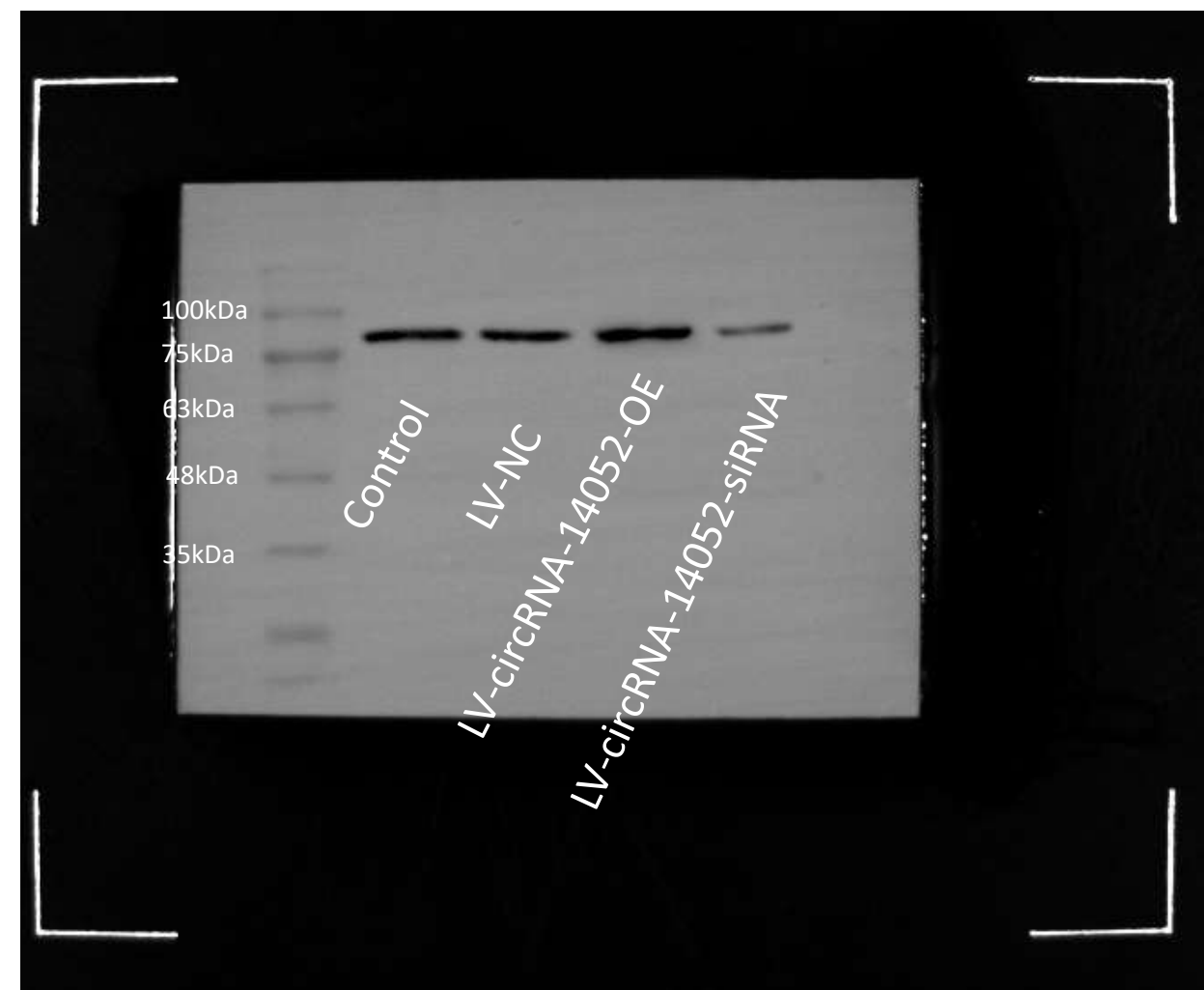

IL-6-1

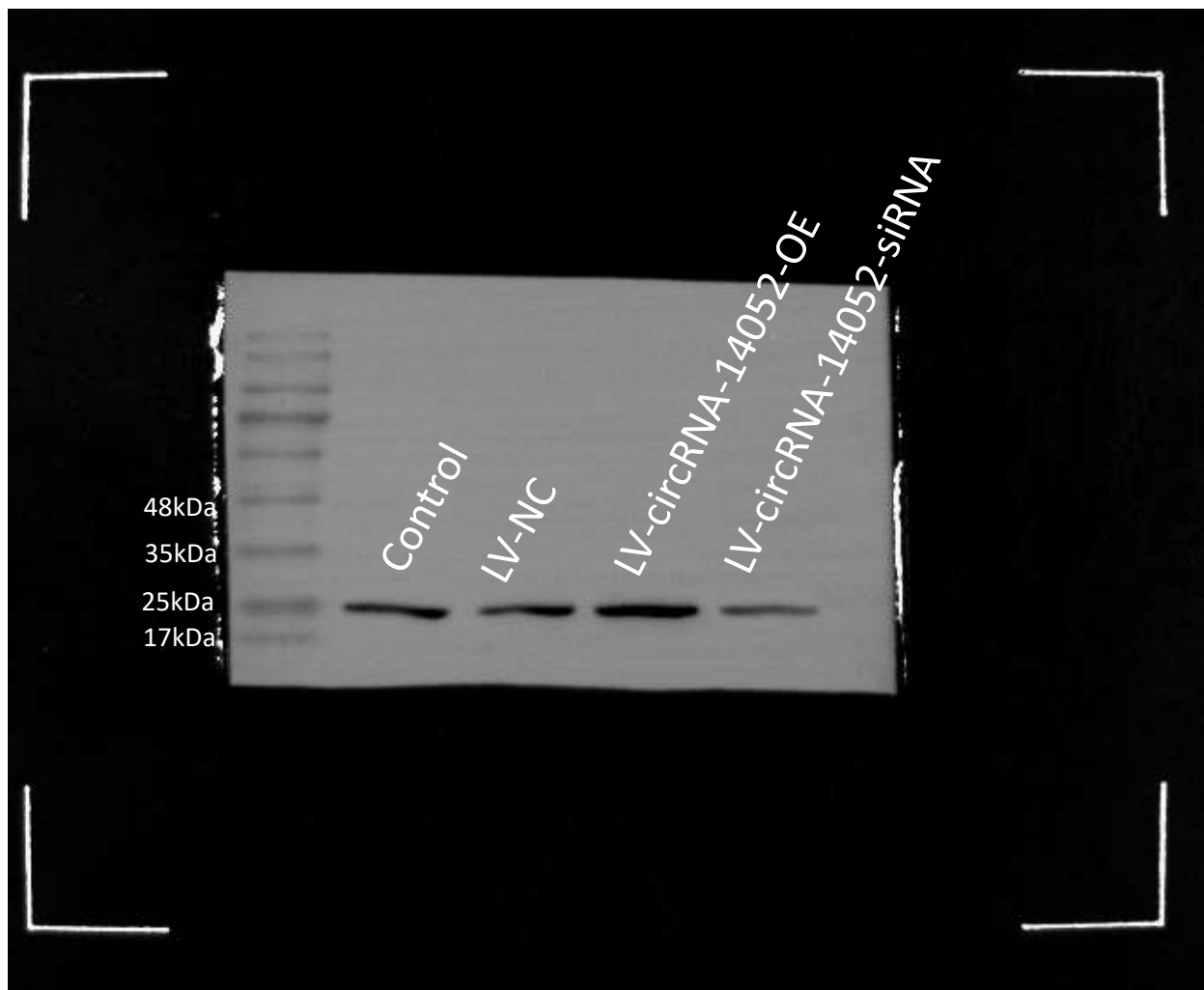

IL-6-2

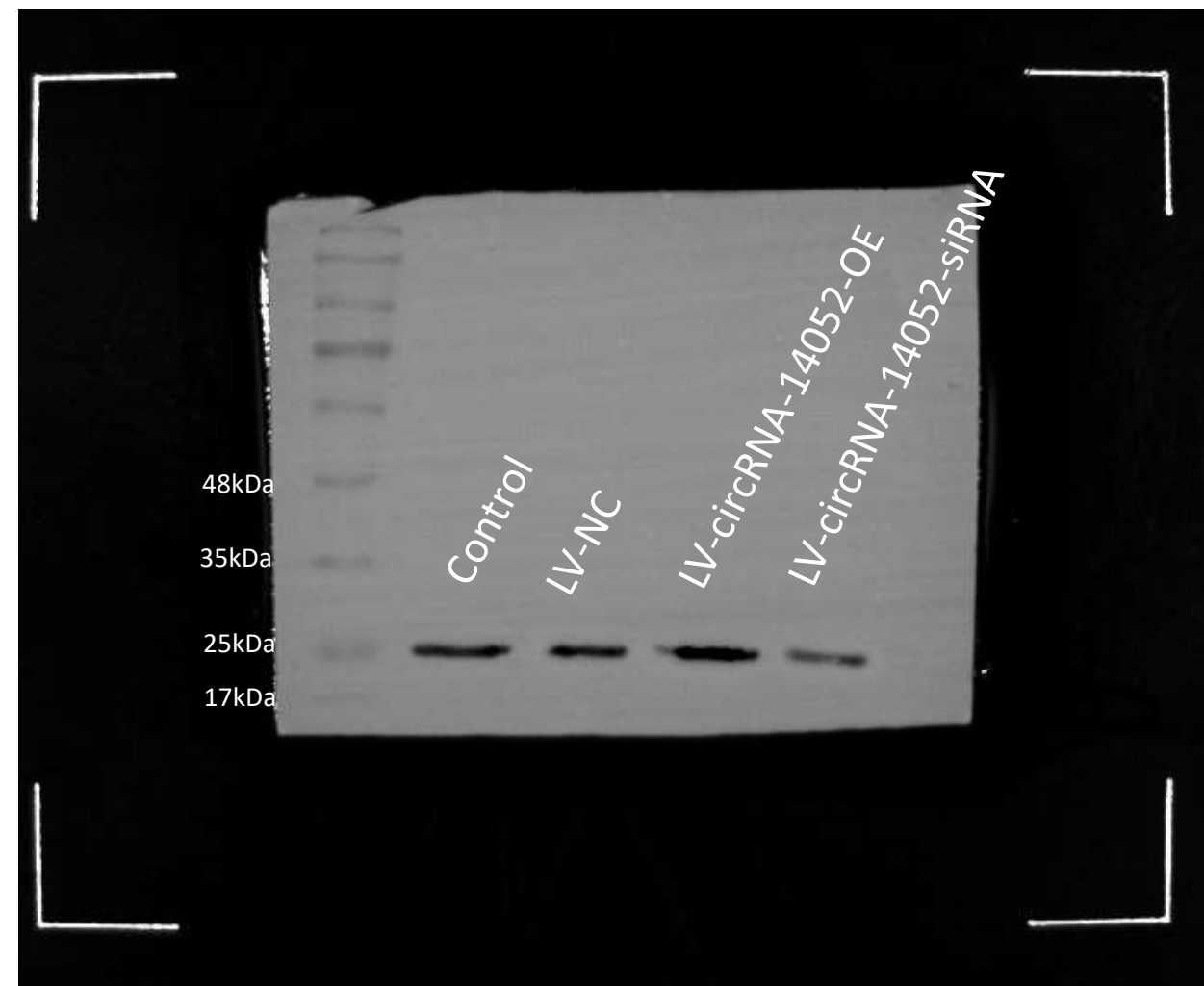

IL-6-3

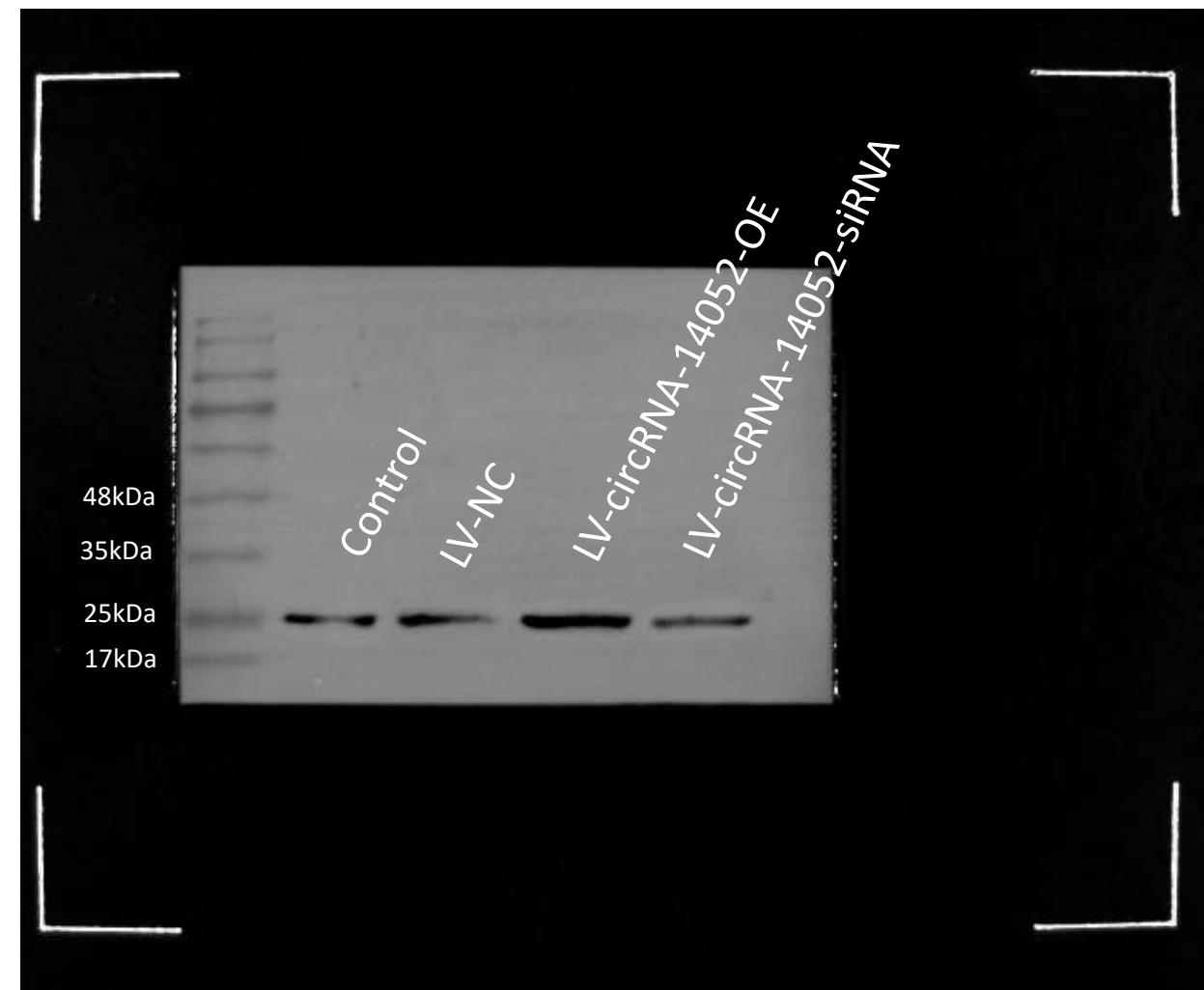

JAK2-1

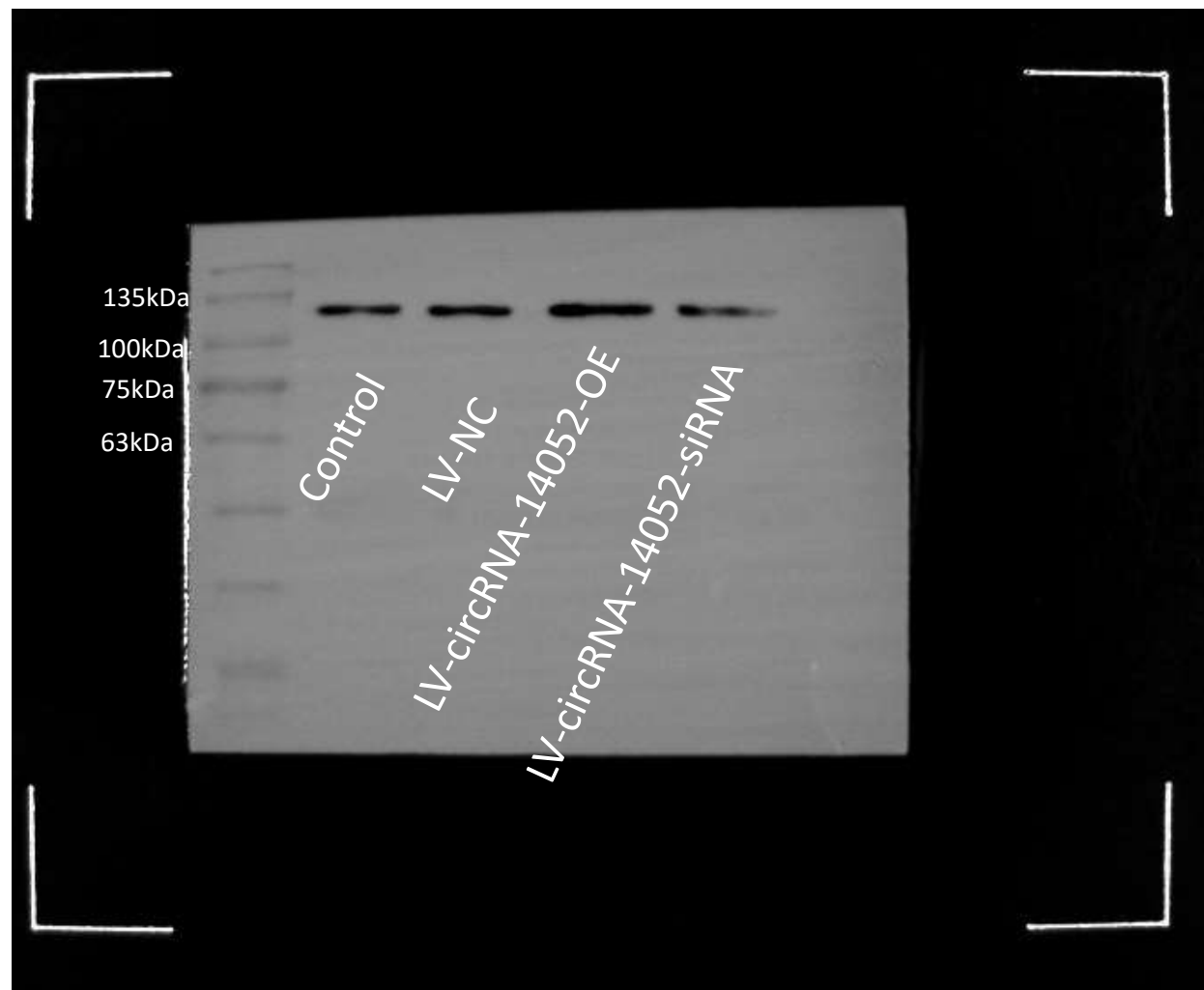

JAK2-2

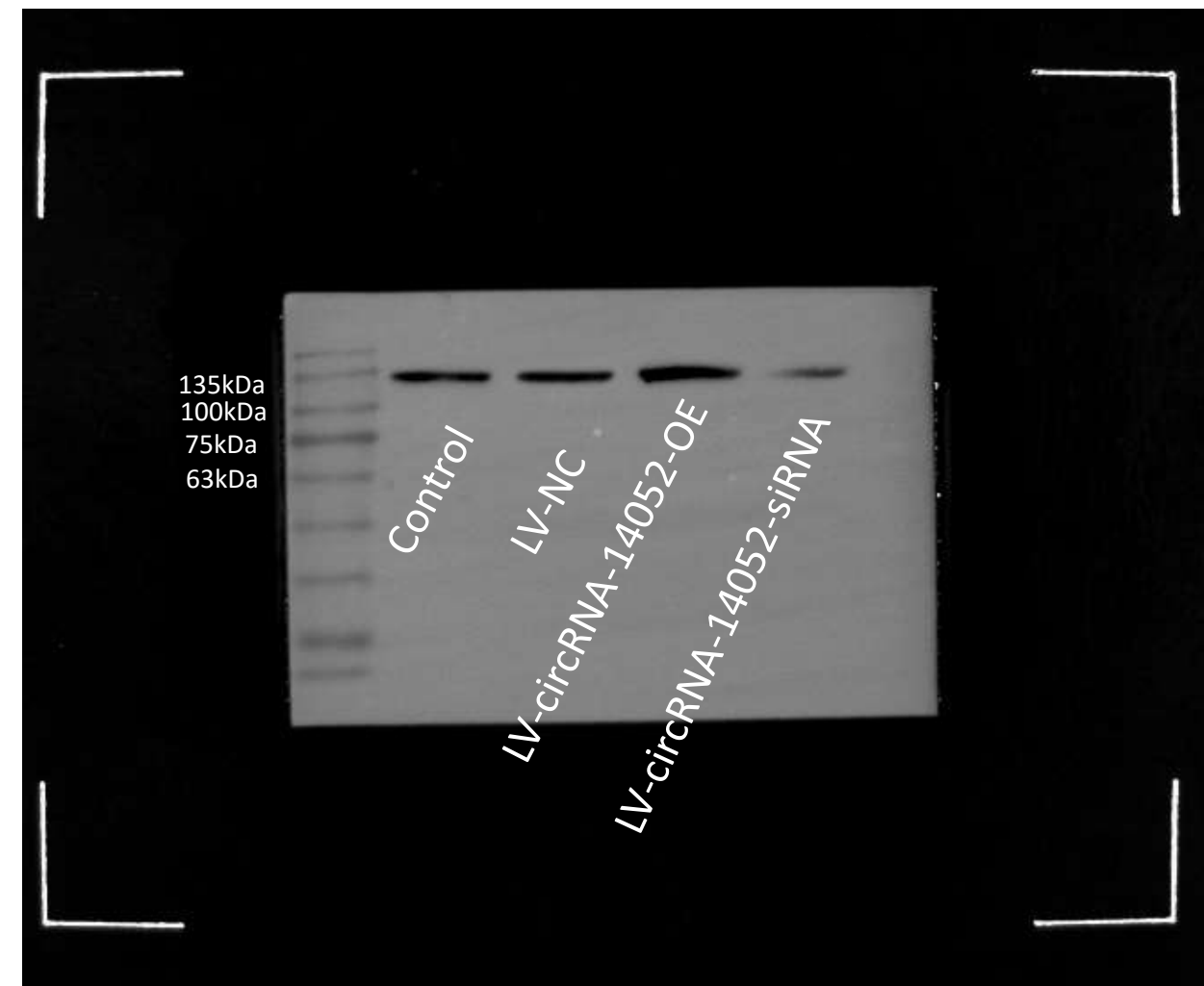

JAK2-3

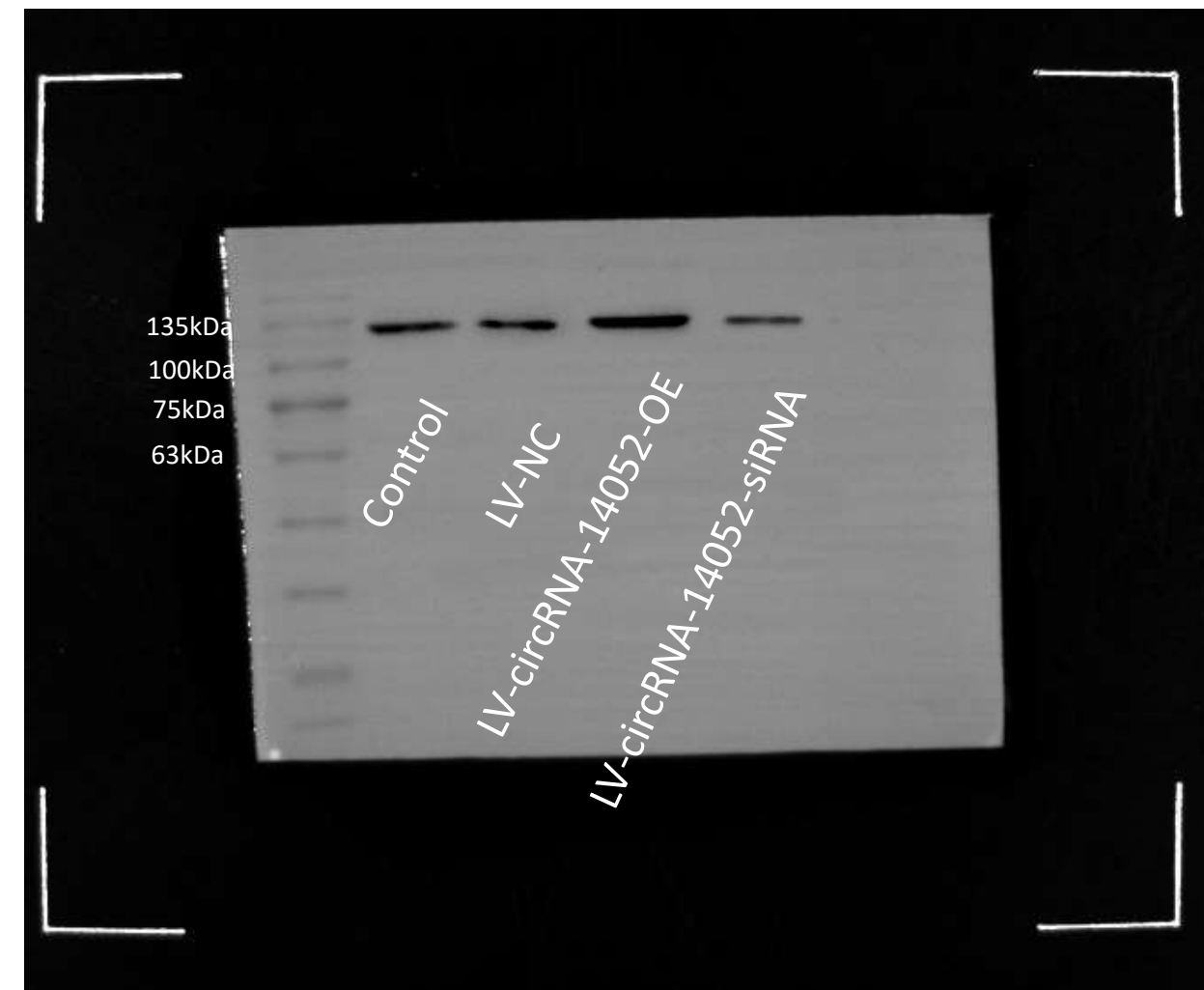

STAT3-1

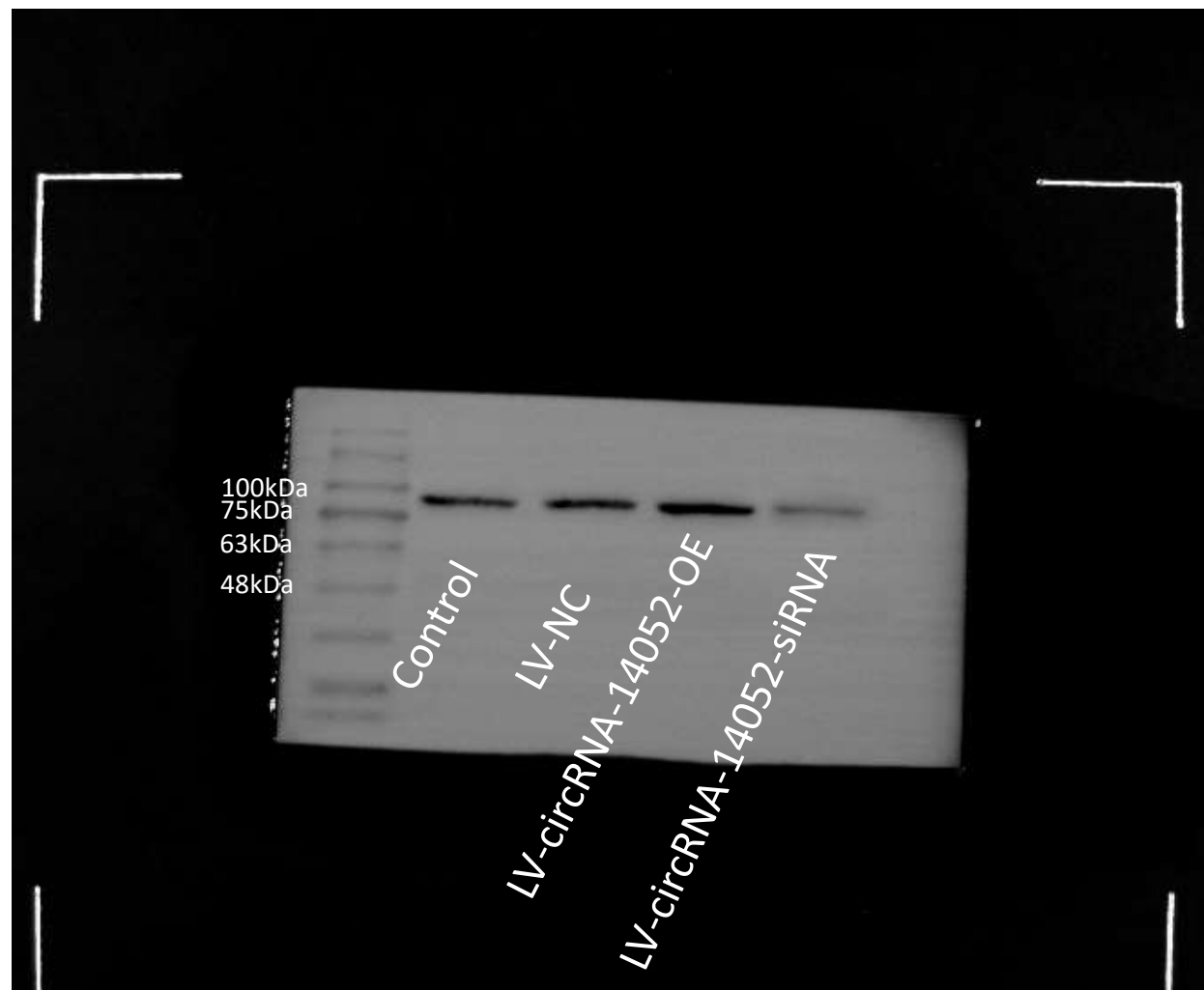

STAT3-2

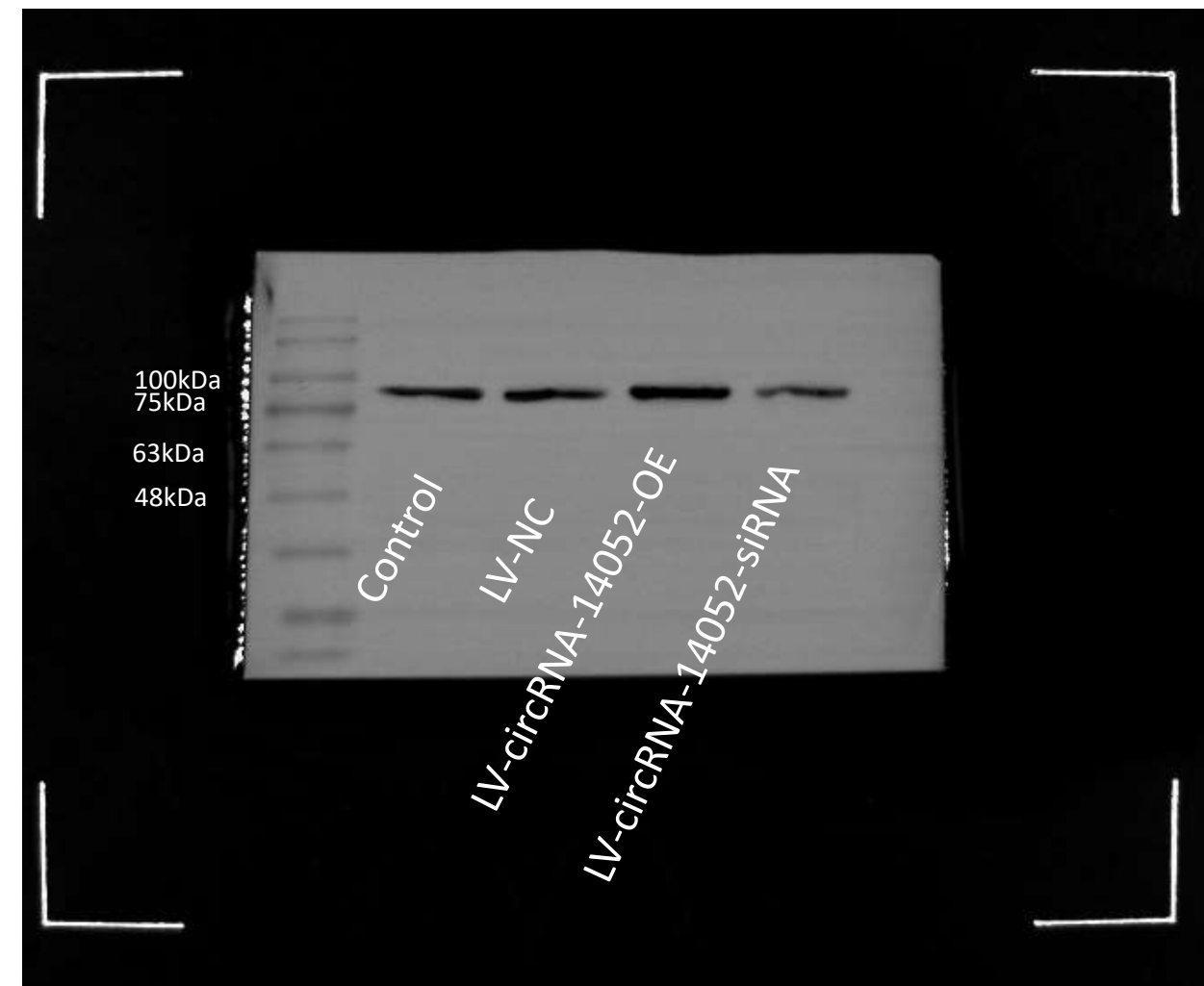

STAT3-3

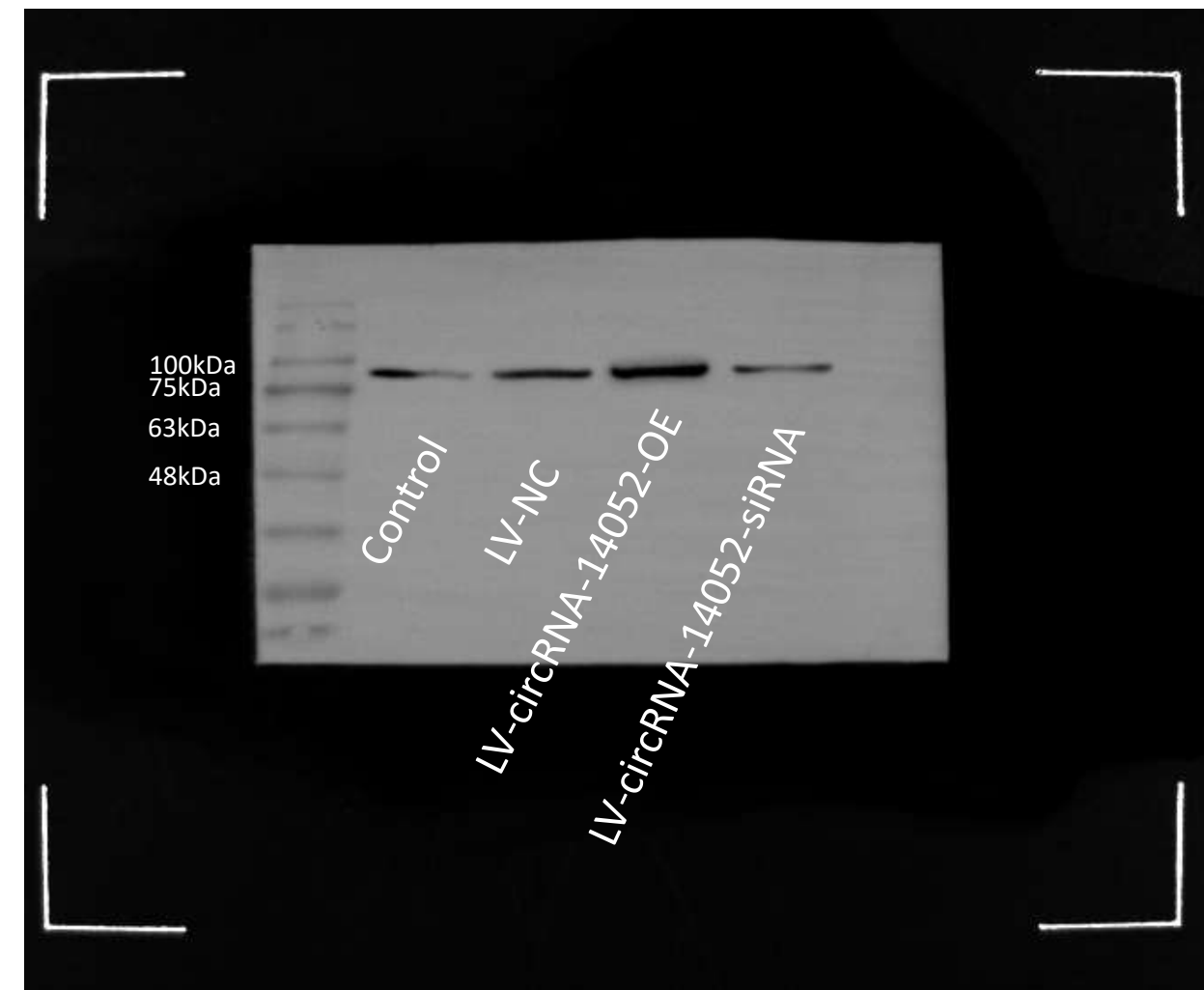

Supplement: Supplementary file 3 — Supplementary Material 3 [file 41065_2025_566_MOESM3_ESM.pdf]
