# Supplementary material for: CircRNA-14052 promotes breast cancer progression via miR-214-3p/IKBKB pathway
Source: Hereditas. 2025 Oct 3;162:202. doi: 10.1186/s41065-025-00566-6 (PMC12495841; doi:10.1186/s41065-025-00566-6)
Supplement: Supplementary file 4 — Supplementary Material 4 [file 41065_2025_566_MOESM4_ESM.docx]

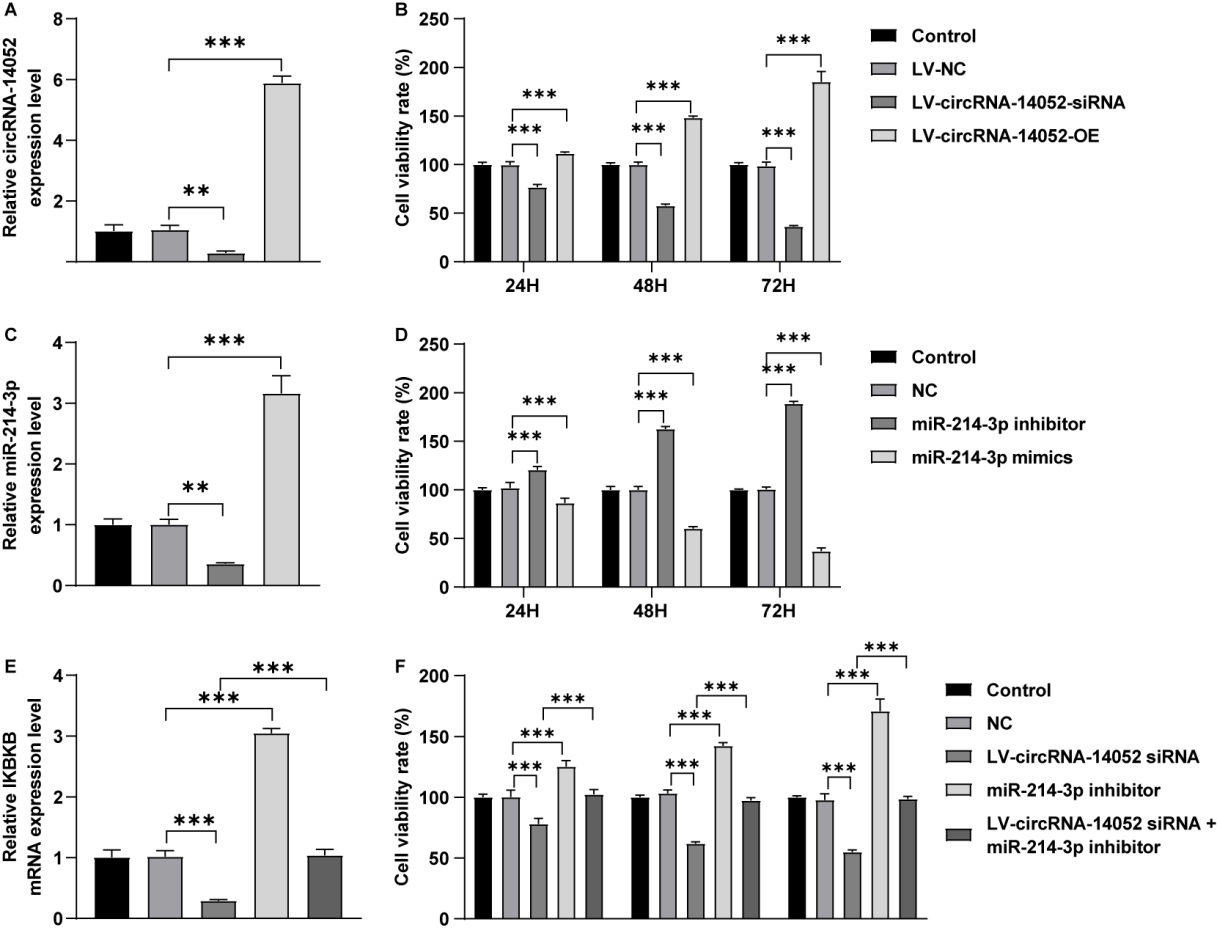


**Figure S1 The circRNA-14052 affects the growth of MDA-MB-231 cells *in vitro* via targeting miR-214-3p/IKBKB axis. (A, B)** MDA-MB-231 cells were transfected with LV-NC, LV-circRNA-14052-siRNA and LV-circRNA-14052-OE. (A) The expression levels of circRNA-14052 in MDA-MB-231 cells were detected by RT-qPCR (n= 3; one-way ANOVA; **P<0.01, ***P<0.001). (B) Cell viability was assessed using the CCK-8 assay (n= 3; two-way ANOVA; ***P<0.001). **(C, D)** MDA-MB-231 cells were transfected with NC, miR-214-3p-mimics or miR-214-3p-inhibitor. (C) The expression levels of miR-214-3p in MDA-MB-231 cells were detected by RT-qPCR (n= 3; one-way ANOVA; **P<0.01, ***P<0.001). (D) Cell viability was evaluated using the CCK-8 assay (n= 3; two-way ANOVA; ***P<0.001). **(E, F)** MDA-MB-231 cells were transfected with LV-NC, LV-circRNA-14052-siRNA, miR-214-3p-inhibitor, and LV-circRNA-14052-siRNA+miR-214-3p-inhibitor. (E) The expression levels of IKBKB in MDA-MB-231 cells were detected by RT-qPCR (n= 3; one-way ANOVA; ***P<0.001). (F) Cell viability was determined using the CCK-8 assay (n= 3; two-way ANOVA; ***P<0.001).
